# One‐Pot Isothermal Nucleic Acid Amplification Assisted CRISPR/Cas Detection Technology: Challenges, Strategies, and Perspectives

**DOI:** 10.1002/advs.202506716

**Published:** 2025-08-30

**Authors:** Hongyan Liu, Hui Yin, Leshan Xiu, Wenyu Wu, Qinqin Hu, Yingchen Xia, Brianna Garcia, Shamsuttiyeba Shifa, Hui Chen, Min Li, Kun Yin

**Affiliations:** ^1^ School of Global Health Chinese Center for Tropical Diseases Research Shanghai Jiao Tong University School of Medicine Shanghai 200025 China; ^2^ School of Public Health Shanghai Jiao Tong University School of Medicine Shanghai 200025 China; ^3^ Department of Thoracic Surgery The First Affiliated Hospital of Shaoyang University Shaoyang 422000 China; ^4^ Department of Thoracic Surgery The First Affiliated Hospital of University of Science and Technology of China (USTC) Division of Life Sciences and Medicine University of Science and Technology of China Hefei 230001 China; ^5^ Department of Chemistry and Biochemistry Texas Tech University Lubbock TX 79409 USA; ^6^ Department of Laboratory Medicine Renji Hospital Shanghai Jiao Tong University School of Medicine Shanghai 200127 China

**Keywords:** CRISPR‐Cas system, diagnosis, isothermal nucleic acid amplification (INA), one‐pot assays, point‐of‐care

## Abstract

The cutting‐edge CRISPR (Clustered Regularly Interspaced Short Palindromic Repeat)/Cas (CRISPR‐associated proteins) system, as an emerging molecular diagnostic technique, is driving revolutionary developments in the detection field due to its high specificity and efficiency. However, the CRISPR‐based assays typically require the combination with an additional pre‐amplification step based on isothermal nucleic acid amplification to meet the requirements of clinical diagnosis, which brings issues including complicated operation and the risk of aerosol contamination. To address these challenges, one‐pot CRISPR platforms are emerging as an attractive solution to streamline workflows, enabling rapid, cost‐effective, and high‐sensitivity diagnostics. This review outlines the current status, challenges, and three key strategies to realize highly efficient one‐pot CRISPR‐based detection. In addition, further perspectives are outlined that will inspire new exploration and promote one‐pot CRISPR/Cas detection as the next generation of diagnostic tools.

## Graphical abstract

This review combines the current status of CRISPR (Clustered Regularly Interspaced Short Palindromic Repeat)‐based molecular diagnostic technology to expound the necessity and challenges faced in promoting the development of one‐pot detection. Besides, the focus is on a comprehensive summary and incisive analysis of emerging strategies for one‐pot CRISPR‐based detection and prospects for future research directions to provide guidance for relevant researchers.

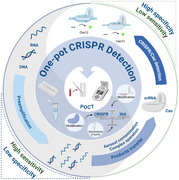

## 1 Introduction

The CRISPR (Clustered Regularly Interspaced Short Palindromic Repeat) system, consisting of CRISPR‐associated proteins (Cas) and CRISPR RNA (crRNA), is a fundamental component of the microbial adaptive immune system.^[^ ^1^ ^]^ It can resist viral infections by recognizing invading nucleic acids and eliminating them through target‐specific endonuclease activity.^[^ ^2^ ^]^ Benefiting from these advantages, CRISPR systems have been widely adopted in molecular diagnostics.^[^ ^3^, ^4^, ^5^ ^]^ Although Cas9 was the first Cas enzyme used in the field of detection, it requires subsequent reactions for signal transduction, which hinders its application.^[^ ^6^ ^]^ Subsequently, the trans‐cleavage mechanism of the Cas12 and Cas13 systems was revealed, providing an approach for direct signal output. Upon recognition of target nucleic acid sequences mediated by crRNA, Cas proteins including Cas12 and Cas13 exhibit nonspecific trans‐cleavage activity that cleaves nearby reporter molecules and generates detectable signals.^[^ ^7^ ^]^ To be more specific, under the guidance of specific crRNA, Cas12 cleaves target double‐stranded DNA (dsDNA) or single‐stranded DNA (ssDNA) while Cas13 cleaves single‐stranded RNA (ssRNA), which catalyzes the indiscriminate cleavage of nearby ssDNA and ssRNA, respectively. Notably, recent studies have demonstrated that Cas12a can also be activated by RNA targets, exhibiting trans‐cleavage activity against both ssRNA and dsDNA with single‐stranded overhangs.^[^ ^8^, ^9^ ^]^ Reporter molecules consisting of ssDNA or ssRNA tagged with a fluorophore and a quencher are introduced to produce a fluorescence signal once they are trans‐cleaved. Among various CRISPR effectors, Cas12 and Cas13 have become the most widely used due to their ability to directly and robustly amplify detection signals through collateral cleavage activity. In contrast, other effectors such as Cas10 and Cas14 are still in the early stages of exploration and have not yet seen broad application in molecular diagnostics. Therefore, this review primarily focuses on recent advances and strategies involving Cas12 and Cas13. Compared to standard PCR‐based tests, CRISPR‐based assays are independent of bulky equipment and have simplified detection procedures and broader accessibility, enabling accurate testing in the field.^[^ ^10^ ^]^ In addition, the rapidly growing CRISPR‐based technology has been utilized for the detection of various types of targets relevant to human health, ranging from typical nucleic acids to small molecules, proteins, and metal ions.^[^ ^11^ ^]^

Despite the high specificity of CRISPR‐based assays for the detection of nucleic acids, their sensitivity, which ranges from picomolar to femtomolar, does not meet clinical requirements.^[^ ^3^ ^]^ To overcome this limitation, various isothermal nucleic acid amplification (INA) techniques, such as loop‐mediated isothermal amplification,^[^ ^12^ ^]^ recombinase polymerase amplification (RPA),^[^ ^13^ ^]^ strand displacement amplification (SDA),^[^ ^14^, ^15^ ^]^ helicase‐dependent isothermal DNA amplification (HDA),^[^ ^16^ ^]^ nucleic acid sequence‐based amplification (NASBA),^[^ ^17^ ^]^ rolling circle amplification (RCA)^[^ ^18^ ^]^ and exponential amplification reaction,^[^ ^19^ ^]^ have been employed to generate sufficient amplicons to trigger CRISPR cleavage for the pre‐amplification of the targets.^[^ ^20^ ^]^ For example, down to 50 copies µL^−1^ of SARS‐CoV‐2 and *E. coli* from clinical samples could be detected by PLACID (Paper‐based LAMP‐CRISPR Integrated Diagnostics).^[^ ^21^ ^]^ In addition, Huang et al. developed a CRISPR‐based assay that combined RPA to enable sensitive detection of SARS‐CoV‐2 within 50 min and achieved a limit of detection (LOD) of 2 copies per sample, which was better than that of other assays in a clinical laboratory.^[^ ^22^ ^]^ By amplifying target sequences, INA methods address the limitation on sensitivity of CRISPR‐based assays, while the high specificity of CRISPR mitigates the risk of false‐positive results often associated with INA‐based detection alone.^[^ ^20^ ^]^ This ideal combination has led to the development of a series of well‐known detection systems, such as SHERLOCK,^[^ ^3^ ^]^ HOLMES,^[^ ^4^ ^]^ and DETECTR,^[^ ^5^ ^]^ which promotes CRISPR‐based detection as a next‐generation candidate for molecular diagnosis.

**Figure 1:**
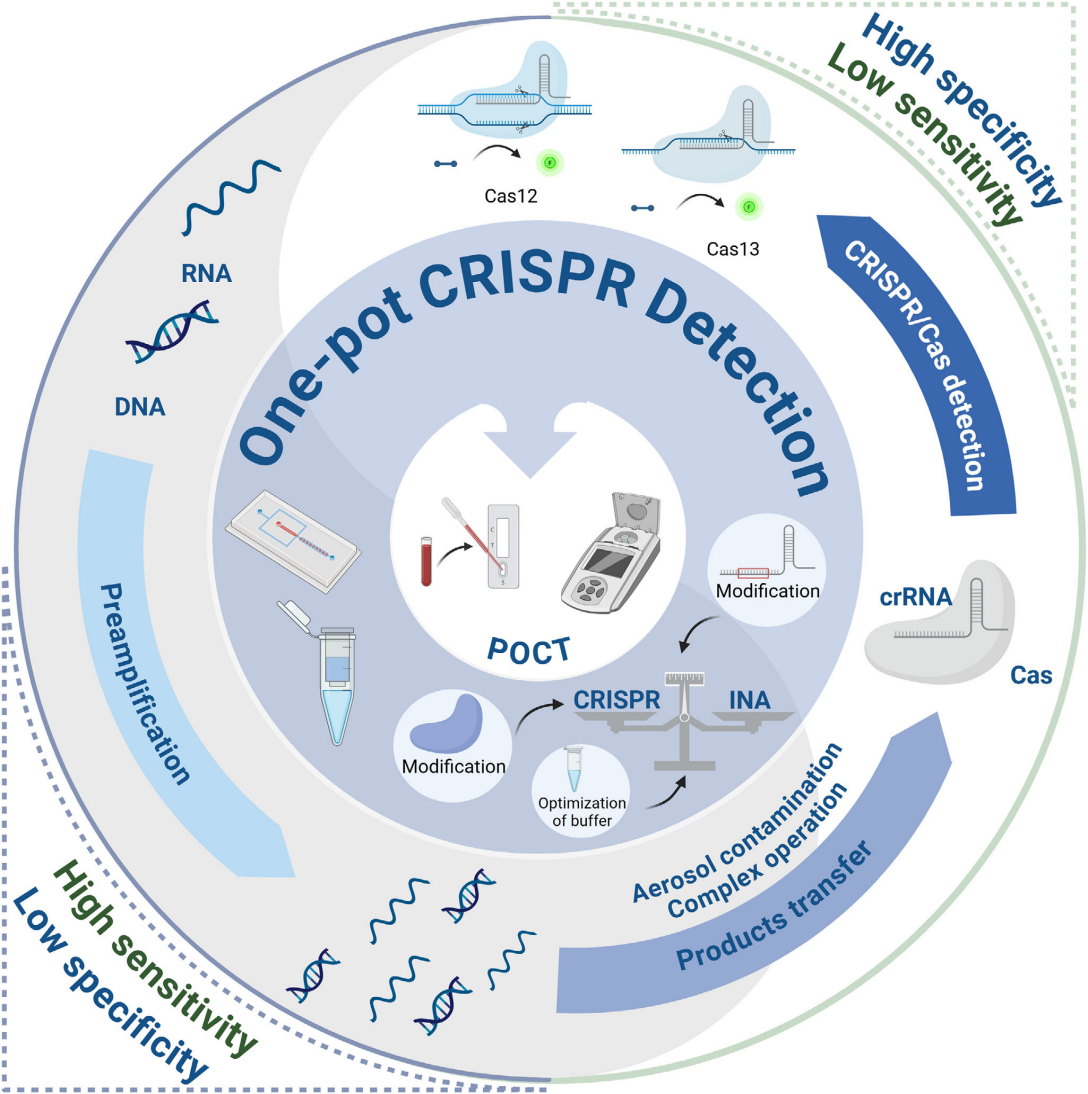
Overview of developing a one‐pot CRISPR/Cas assay. The coupling of CRISPR and INA offers complementary advantages to improve performance, but the two‐step approach leads to contamination and complex operations, emphasizing the necessity of developing a one‐pot assay. Improving the biocompatibility between INA and CRISPR is the main research object of the one‐pot strategy, promoting POCT.

The combination of the CRISPR‐Cas system and INA can improve the analytical performance of clinical diagnosis, but the multi‐step operation increases complexity, prolongs response time, and carries an increased risk of cross‐contamination, seriously impeding the commercialization and application in point‐of‐care testing (POCT). To overcome these limitations, “one‐pot” CRISPR detection strategies are developed by integrating INA and CRISPR detection within a sealed container, which is the evolution of the traditional two‐step INA‐CRISPR workflow, not a complete departure from the use of INA (**Figure** **1**).

One‐pot CRISPR‐based detection allows for a streamlined, user‐friendly approach that significantly reduces the risk of contamination and eliminates the necessity for sequential reaction steps, enabling more rapid and straightforward testing to fulfill POCT. For example, the conventional two‐step SHERLOCK assay can achieve a detection sensitivity of the zeptomolar level with 30–60 min, requiring separate amplification and multiple manual steps such as tube opening and liquid transfer. By comparison, despite only offering the sensitivity of femto‐ to attomolar range, the one‐pot SHERLOCK assay can shorten the detection time to 15–30 min without liquid transfer, which was significantly less time‐ and labor‐intensive than the two‐step SHERLOCK.^[^ ^23^ ^]^ In addition, one‐pot strategy overcomes the limitations of quantifying original target concentration, which often arise from amplification biases and inaccurate product transfer during two‐step procedures. Despite the simplified procedure and lower cross‐contamination risk, the one‐pot assays still confront several challenges, particularly in increasing the biocompatibility by balancing the reaction dynamics of INA and CRISPR processes, which usually compromise the detection sensitivity compared with the two‐step approaches. For instance, in the RPA‐CRISPR/Cas12a system, the target nucleic acid is not only the template of RPA but also can be cis‐cleaved by the CRISPR, reducing the detection efficiency due to reaction competition and template degradation.^[^ ^24^ ^]^

To address the above challenges, several strategies have been proposed to develop robust, rapid, and uncomplicated one‐pot CRISPR detection methods, including setting physical separation within a reaction vessel,^[^ ^25^ ^]^ optimizing reaction buffer,^[^ ^26^ ^]^ regulating CRISPR activity,^[^ ^27^ ^]^ and utilizing integrated devices.^[^ ^28^ ^]^

This review summarizes recent advances in one‐pot CRISPR‐based detection systems, highlighting current strategies, challenges, and future perspectives. By addressing these challenges, one‐pot CRISPR platforms can play a significant role in the development of more convenient, rapid, and accurate diagnostic tools, which are essential for widespread clinical and field applications. Although numerous reviews have discussed CRISPR/Cas‐based diagnostics, they typically focus on specific technical challenges (such as amplification‐free,^[^ ^29^ ^]^ multiplex^[^ ^30^ ^]^ or non‐nucleic acid detection^[^ ^31^ ^]^) or emphasize application scenarios (including diagnosis of infectious diseases^[^ ^32^ ^]^ and cancer,^[^ ^33^ ^]^ and prenatal screening^[^ ^34^ ^]^) for one or all Cas effectors. In contrast, this review uniquely focuses on the challenge of integrating INA with CRISPR in a single reaction system and systematically evaluates the strategies designed to overcome the resulting incompatibilities. Compared with the only two existing reviews that explicitly discuss one‐pot CRISPR detection systems,^[^ ^35^, ^36^ ^]^ several points make differences, our review 1) systematically dissects the key incompatibility issues, 2) provides a detailed comparative analysis of emerging design strategies aimed at overcoming these technical bottlenecks, and 3) offers forward‐looking perspectives on future integration with artificial intelligence (AI)‐driven data interpretation and portable device miniaturization to facilitate the transition of one‐pot CRISPR diagnostics into real‐world settings.

## 2 Challenge

While CRISPR detection systems can approximately reach a detection sensitivity of the femtomolar level, this falls short of the requirements for clinical diagnostics. Integrating INA with CRISPR significantly enhances sensitivity,^[^ ^37^ ^]^ making it a cutting‐edge approach for diagnostic applications. However, two‐step methods combining INA and CRISPR detection are associated with complex procedures and aerosol contamination risk.^[^ ^38^, ^39^ ^]^ To address these limitations, researchers are actively exploring one‐pot systems, which streamline the process by combining CRISPR and INA in a single reaction.^[^ ^40^, ^41^, ^42^ ^]^ Initially, directly mixing INA and CRISPR components in one pot was operated to avoid opening the reaction vessel between steps, such as HOLMESv2,^[^ ^43^ ^]^ iSCAN‐OP^[^ ^44^ ^]^ and CRISPR‐top.^[^ ^37^ ^]^ Despite promising advances in mitigating contamination risks, substantial challenges remain in achieving efficient, reliable one‐pot CRISPR detection due to lower sensitivity compared to their two‐step counterparts.

**Figure 2:**
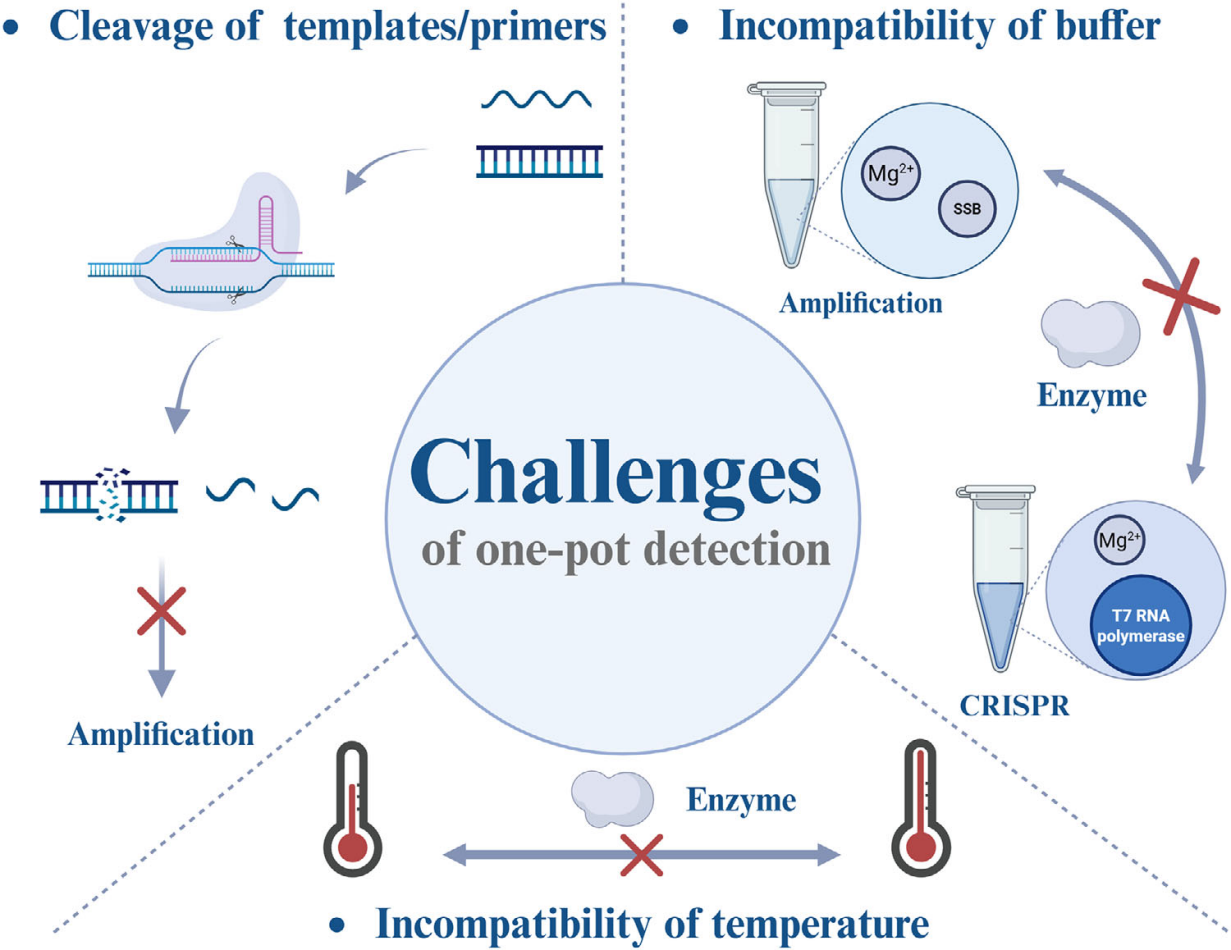
Challenges of one‐pot CRISPR/Cas detection. Three major challenges remain in the development of a high‐sensitive one‐pot detection, including the cleavage of templates and primers by CRISPR restraining the amplification leading to reduction of amplicons, incompatibility of buffer and temperature inhibiting the activity of the enzyme thus diminishing the efficiency of INA and CRISPR.

The main obstacle lies in the inherent incompatibilities between the two systems of INA and CRISPR, particularly reagents and temperature conditions. In this section, these challenges are categorized into three main types according to the incompatible objects: cleavage of templates and primers of amplification, incompatibility of buffer and temperature (**Figure** **2**).

### 2.1 Cleavage of Templates and Primers of Amplification

Mixing INA and CRISPR components in a co‐existing space introduces complex interactions impairing amplification efficiency, which is the key factor resulting in low sensitivity. Specifically, the cis‐ and trans‐cleavage activities of CRISPR enzymes may degrade both target nucleic acids and INA‐related primers, thereby reducing the amplicon yield.^[^ ^45^, ^46^, ^47^ ^]^

For example, in one‐pot RPA‐CRISPR/Cas12a systems, the cis‐cleavage activity of Cas12a will cleave dsDNA templates that serve as substrates for RPA,^[^ ^48^ ^]^ while its trans‐cleavage activity may degrade RPA primers non‐specifically, further hindering the amplification process.^[^ ^24^ ^]^ CRISPR‐based RNA detection systems experience similar problems, for instance, when reverse transcription (RT)‐LAMP is used in a one‐pot assay to detect viral RNA, Cas12b degrades the initial LAMP products, resulting in weak amplification signals observed by gel electrophoresis.^[^ ^49^ ^]^ Similarly, the T7 RNA polymerase in the CRISPR/Cas13a system can transcribe DNA amplicons into RNA, reducing the efficiency of nucleic acid amplification.^[^ ^50^ ^]^

Due to these factors, direct mixing of INA and CRISPR systems often results in lower sensitivity and longer response times compared to the two‐step methods. One study reported that a one‐pot CRISPR assay combining LAMP and CRISPR in a simultaneous reaction format exhibited decreased sensitivity for detecting *Pseudomonas aeruginosa* and required more than one hour to complete the detection process.^[^ ^51^ ^]^

### 2.2 Incompatibility of Buffer

Whether it is INA or CRISPR, buffer is an important condition for the reaction. However, the difference in composition of buffer between INA and CRISPR brings the issue of incompatibility of buffer, introducing complex reagent composition in one‐pot detection, which will cause mutual inhibition between the two systems. A mixed buffer may be incapable of providing the optimal pH for each reaction in one pot. Furthermore, partial INA technologies involve multiple enzymes and cofactors, which is possible to give rise to unexpected interactions with CRISPR components, resulting in reduced efficiency.^[^ ^12^, ^13^ ^]^ For example, as a critical component of RT‐recombinase‐aided amplification (RAA), the ssDNA‐binding protein that was designed to bind to the RT‐RAA primers, could also bind to the crRNA, resulting in obstacles to crRNA function in the one‐pot detection.^[^ ^52^, ^53^, ^54^ ^]^ In addition, reagents required in both two systems, such as Mg^2+^, can cause kinetic imbalances due to competitive reactions. For example, Wang et al. found Mg^2+^ in the CRISPR/Cas buffer could hinder the RPA performance by influencing the activity of the polymerase system.^[^ ^25^ ^]^ Besides, studies have confirmed the emphasis on Mg^2+^ concentration in the fine performance of CRISPR.^[^ ^55^ ^]^ Accordingly, optimization of the concentration of some reagents is commonly operated in one‐pot CRISPR‐based detection.

### 2.3 Incompatibility of Temperature

Another key challenge in the path of developing one‐pot assays is temperature incompatibility. CRISPR systems typically use Cas enzymes derived from mesophilic organisms, such as Cas12a and Cas13a, which operate optimally ≈37 °C, while some INA techniques require higher temperatures.^[^ ^56^ ^]^ In particular, Cas12b, which is derived from thermotolerant bacteria, can work at 37–65 °C. The operating temperature range of different INA is narrow. For example, the commonly used LAMP and RPA show the best efficiency at 55–65^[^ ^12^ ^]^ and 35–42 °C,^[^ ^13^ ^]^ respectively. Other INA technologies, such as HDA and SDA, have evolved to work at ≈60 °C after the update.^[^ ^14^, ^15^, ^16^, ^57^, ^58^ ^]^

The incompatibility of temperature limits the choice of INA and Cas, which reduces the possibility of adaptation. Ding et al. made some attempts to balance the temperature of CRISPR/Cas12a and LAMP.^[^ ^59^ ^]^ Low Mg^2+^ concentration is beneficial in reducing amplification temperature, while the cleavage of Cas12a depends on high Mg^2+^ concentration.^[^ ^59^, ^60^, ^61^ ^]^ Therefore, they optimized the concentration of Mg^2+^ and the pyrophosphatase that was used to degrade the byproduct magnesium pyrophosphate for releasing free Mg^2+^. In addition, the phosphorothioated inner primers were employed to further reduce the operation temperature of LAMP. However, the measures involved complex composition optimization and low generalizability, limiting the choice of Cas and INA. Therefore, developing thermostable Cas enzymes is of great significance for expanding the choice of INA and Cas in one‐pot detection.

## 3 Strategies of One‐Pot CRISPR/Cas Assays

**Figure 3:**
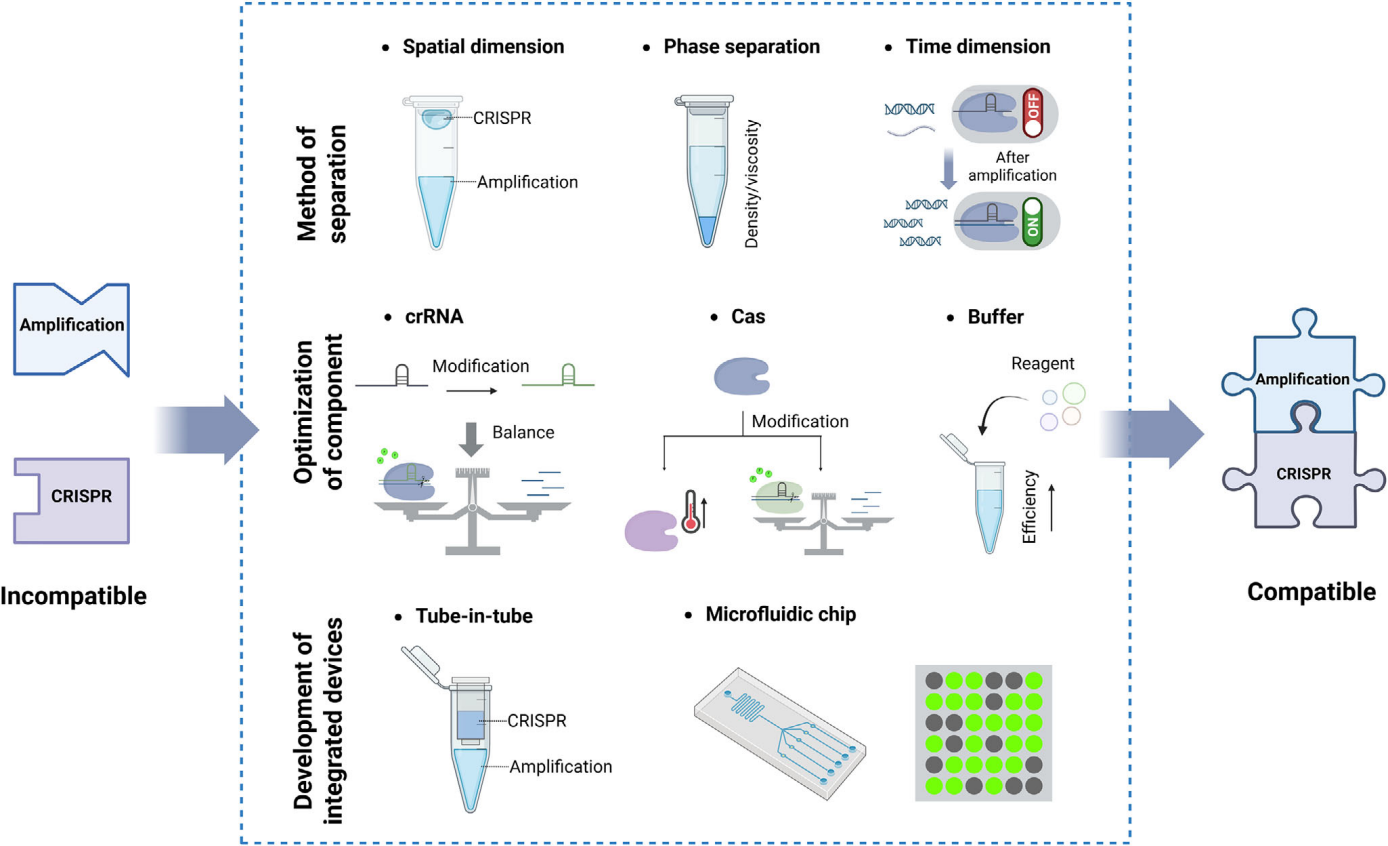
Schematic overview of strategies for one‐pot CRISPR/Cas detection. The incompatibility between INA and CRISPR was addressed through three main strategies, including the method of separation, optimization of components, and development of integrated devices. More detailed implementation methods of these strategies included separation in spatial and time dimensions, phase separation, modification of crRNA and Cas, optimization of buffer, tube‐in‐tube vessel, and microfluidic chip.

Integrating both reactions into a single tube minimizes contamination risks from opening lids but necessitates addressing the inherent incompatibility between INA and CRISPR systems. Encouragingly, researchers have constructed various promising strategies to deal with the above challenges for one‐pot CRISPR detection. Physical barriers^[^ ^41^ ^]^ have been explored to physically separate the two systems within one tube to prevent the mutual influence between the INA and CRISPR systems. Additionally, some integrated devices have also been developed to enable integration within a sealed vessel, but these solutions often require extra centrifugation or mixing steps, reducing their suitability for POCT.^[^ ^28^, ^37^, ^62^ ^]^ Owing to allowing automatically and slowly dynamic connection between two systems, phase‐separation methods eliminate extra operations, but still yield lower sensitivity compared to the two‐step approaches.^[^ ^63^ ^]^ To further simplify workflows while keeping performance, some strategies have been developed to encourage early accumulation of amplification products before CRISPR activation.^[^ ^64^, ^65^ ^]^ Currently, these strategies focus on reducing crRNA activity to delay the CRISPR reaction, allowing the amplification phase to proceed unhindered, which has been achieved using suboptimal protospacer adjacent motifs (PAMs) or modified crRNAs.^[^ ^64^ ^]^ Despite simplifying workflows, these strategies will compromise the efficiency of the CRISPR system.^[^ ^66^ ^]^ An innovative solution is time‐based isolation strategies, such as light‐activated crRNA, allowing the CRISPR reaction to occur only after sufficient amplification has completed.^[^ ^24^, ^42^ ^]^ In addition, other strategies have also been developed to realize the one‐pot detection, such as the modification of Cas protein to adjust cleavage kinetics for balance between amplification and CRISPR,^[^ ^40^ ^]^ the development of thermostable enzymes to dispose of the incompatibility of temperature,^[^ ^4^, ^67^, ^68^ ^]^ and the addition of reagents to directly or indirectly improve sensitivity (**Table** **1**).^[^ ^69^, ^70^, ^71^ ^]^ The overview of one‐pot CRISPR‐based detection strategies is presented in **Figure** **3**.

**Table 1:** Overview of the one‐pot CRISPR/Cas detection strategies.

Strategy	Mechanism	Platform	Method	Target	Sensitivity	Temperature	Nucleic acid extraction	Time	Quantification	Advantage	Limitation	Ref.
Method of separation	Isolation in spatial dimension	Cas12aVDet	Cas12a + RPA	Mycoplasma	10 aM	37 °C	Yes	30 min	No	Without complexed design, low cost, rapid.	Complex mixing steps.	[25]
		opvCRISPR	Cas12a + RT‐LAMP	SARS‐CoV‐2	5 copies reaction^−1^	65 °C + 37 °C	Yes	45 min	No		Complex mixing steps and temperature change.	[72]
		One‐pot‐RPA‐CRISPR/Cas12a	Cas12a + RPA	Respiratory pathogens	2.5 copies µL^−1^	37 °C + 55 °C	Yes	93 min	No	Without complexed design, low cost.		[41]
	Phase separation	DAMR	Cas12a + RPA	HPV ^a)^ 16, HPV 18	10 copies reaction^−1^, 100 copies reaction^−1^	37 °C	No	60 min	Yes	Without an extra mixing step, low cost.	High time cost and impressionable reproducibility	[73]
		Glycerol‐Enhanced One‐Pot RPA‐CRISPR/Cas12a	Cas12a + RPA	ASFV ^b)^ and SARS‐CoV‐2	35.93 Ct, 10 copies reaction^−1^	37 °C	Yes		No			[74]
	Isolation in the time dimension	Photocontrolled One‐Pot RPA‐CRISPR‐Cas12a DNA Assay	Cas12a + RPA	SARS‐CoV‐2	10 copies reaction^−1^	37 °C	Yes	30 min	No	Simple and rapid	Complex optimization of the ratio of PC ^c)^‐linker‐containing oligonucleotides to crRNA.	[24]
		Light‐start CRISPR‐Cas12a reaction with caged crRNA	Cas12a + RPA	SARS‐CoV‐2	1 copies µL^−1^	37 °C	Yes	40 min	No		Low universality, complex evaluation of the position for NPOM ^d)^ modification.	[42]
		POIROT	Cas12a + RPA	SARS‐CoV‐2, HPV 16, HPV 18	1 copies µL^−1^	37 °C	Yes	20 min	No	Rapid, universal, not affected by crRNA sequence.	Cas12a enzyme activity may be damaged.	[66]
		RNA‐based CHA circuit with CRISPR‐Cas12a for one‐pot detection of miRNAs	Cas12a + CHA	Let‐7a	81.96 fM	37 °C	Yes	120 min	Yes	Detecting miRNA, small molecules, and proteins by integrating RNA aptamer sequences.	Complex design.	[75]
Optimization of the component	Modification of crRNA	sPAMC	Cas12a + RPA	SARS‐CoV‐2	2.34 fM	37 or 42 °C	Yes	15 min	No	Fast, high reliability, and flexibility.	Damaging the efficiency of CRISPR, requiring to tailor for a specific target sequence.	[64]
		AIOD‐CRISPR	Cas12a + RPA	SARS‐CoV‐2	3 copies reaction^−1^	37 °C	Yes	20 min	No	Not restricted by the PAM.	Requiring to tailor for a specific target sequence.	[65]
	Modification of the Cas protein	CRISPR‐SPADE	Cas12b + RT‐LAMP	Alpha, Beta, Gamma, Delta, Omicron, and the universal N gene of SARS‐CoV‐2	25/50/500/12/15/15 copies µL^−1^	62 °C	Yes	10–30 min	No	Fast, suitable for LAMP.	Complex characterization and engineering of Cas.	[67]
		One‐pot testing using suboptimal PAMs of Cas12b	Cas12b + LAMP	SARS‐CoV‐2	0.5 copies µL^−1^	61 °C	Yes	30 min	No	High universality, suitable for LAMP.		[40]
	Optimization of reaction buffer	HBV HOLMESv2	Cas12b + LAMP	HBV ^e)^	25 copies mL^−1^	60 °C	Yes	<60 min	Yes	Fast, simple, low cost.	Optimization of the dosage of additives.	[76]
	Template protection	REVERSE	Cas13a + RT‐RPA	SARS‐CoV‐2	0.5 copies µL^−1^	42 °C	Yes	30 min	No		Limited scope of application.	[40]
		OAR‐CRISPR	Cas12b + RPA	BFB ^f)^, genetically modified soybean DBN9004	30 copies µL^−1^	40 °C	Yes	24 min	Yes	Without the PAM requirement.	Complex primer optimization.	[77]
Development of integrated devices	Tube‐in‐tube vessel	ECS‐CRISPR	Cas13a + RPA	ASFV, SARS‐CoV‐2	3 copies µL^−1^	39 °C	Yes	25 min	No	Not limited by liquid volume, rapid.	Complexed design and production, mixing steps, high cost.	[37]
		STAR	Cas12a + RPA	Salmonella typhimurium	100 CFU mL^−1^	37 °C	Yes	90 min	Yes	Without mixing steps.	Time‐consuming, complexed design and production, high cost.	[78]
	Microfluidic chip	LOC‐CRISPR	Cas12a + RPA	IVA ^g)^, IVB ^h)^, HRSV ^i)^, three variants of SARS‐CoV‐2	100 copies mL^−1^	42 °C	Yes	<60 min	No	Suitable for POCT, simple operation, automatic.	Complexed design and production, high cost.	[28]
		MEDICA	Cas13a + RPA	HPV 16, HPV 18	5 copies µL^−1^	37 °C	Yes	25 min	Yes		Complexed optimization of buffer.	[79]

^a)^ Human papillomavirus;

^b)^ African swine fever virus;

^c)^ Photocleavable;

^d)^ 6‐nitropiperonyloxymethyl‐caged thymidine;

^e)^ Hepatitis B virus;

^f)^ Bacterial fruit blotch;

^g)^ Influenza A virus;

^h)^ Influenza B virus;

^i)^ Human respiratory syncytial virus.

### 3.1 Method of Separation

#### 3.1.1 Isolation in Spatial Dimension

**Figure 4:**
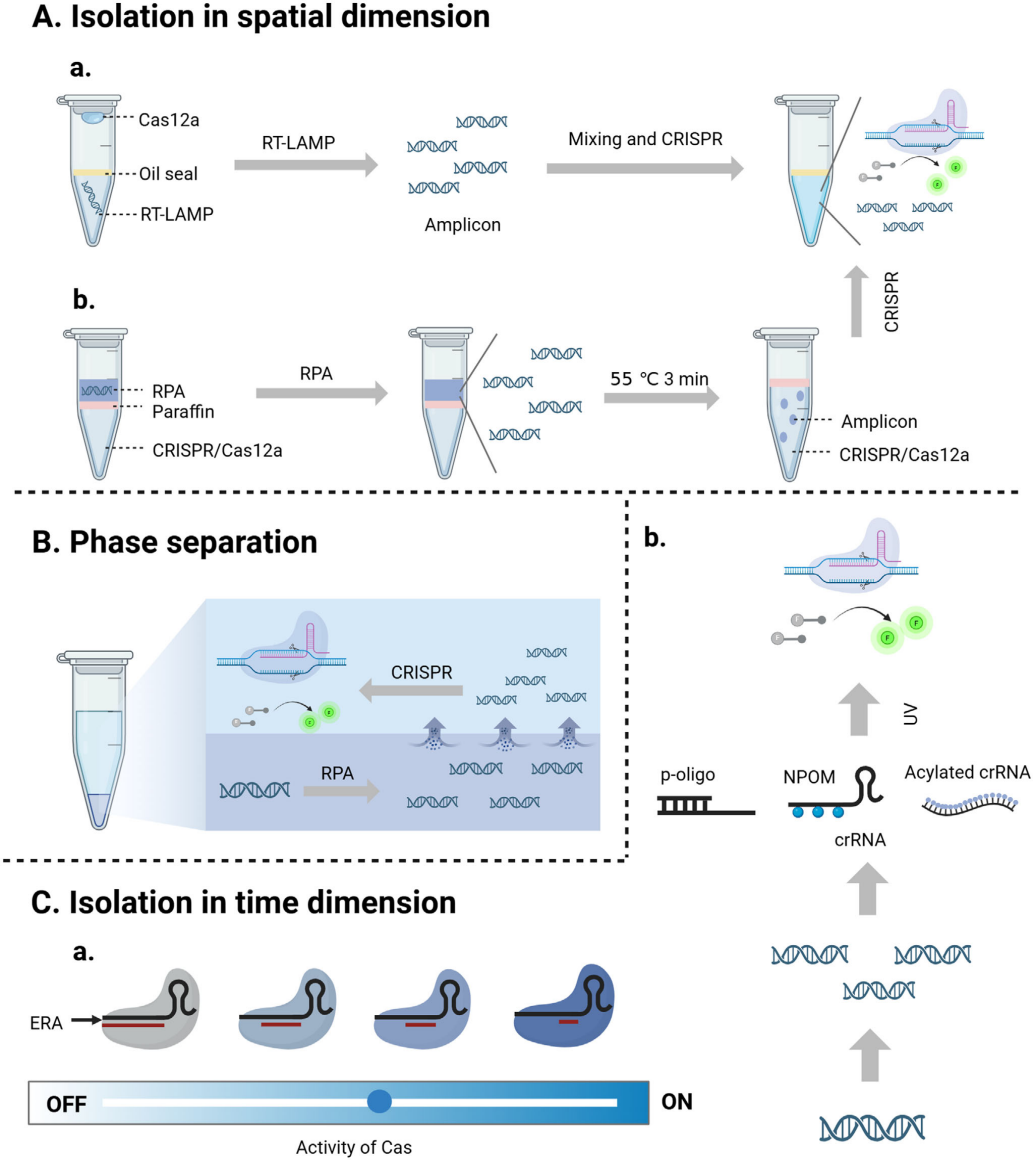
Schematic illustration of the method of separation. A) Isolation in spatial dimension. a) The platform named opvCRISPR temporarily stored CRISPR reagent in the tube cap to physically separate with the amplification system.^[^ ^72^ ^]^ b) One‐pot‐RPA‐CRISPR/Cas12a physically separated two systems with paraffin.^[^ ^41^ ^]^ B) Phase separation. Sucrose was added to establish a DAMR system to separate amplification and CRISPR.^[^ ^73^ ^]^ C) Isolation in a temporal dimension. a) ERAs controlled the activity of Cas to regulate the CRISPR detection.^[^ ^27^ ^]^ b) The crRNA was silenced by p‐oligo,^[^ ^24^ ^]^ NPOM,^[^ ^42^ ^]^ or 2′‐hydroxyl group acylation to protect amplification, recovering CRISPR activity by UV after amplification.^[^ ^66^ ^]^

To minimize interactions between INA and CRISPR while avoiding opening the lid, the air column and oil phase are employed to physically separate the CRISPR system from INA at the bottom of the tube. For example, Wang et al. introduced Cas12aVDet, a one‐pot visual detection method wherein all reagents were placed at the tube bottom, while Cas was localized on the tube wall. Following a 15‐minute RPA reaction, the Cas12a enzyme was centrifuged into the reaction mix for target cleavage.^[^ ^25^ ^]^ Subsequently, a one‐pot visual RT‐LAMP‐CRISPR (opvCRISPR) method was developed by the same team for SARS‐CoV‐2 detection, in which CRISPR reagents were added on the lid and centrifuged after amplification completed for high‐sensitivity detection (**Figure** **4**A).^[^ ^72^ ^]^ There are many similar methods adopting this setup, such as ORCas12a‐BRVc,^[^ ^80^ ^]^ OCTOPUS,^[^ ^81^ ^]^ RAVI‐CRISPR,^[^ ^82^ ^]^ ICB‐LAMP‐CRISPR/Cas12a,^[^ ^83^ ^]^ and CRISPR/Cas12a‐E‐LAMP.^[^ ^84^ ^]^

Paraffin, with less density than water, can separate or mix different phases by utilizing its melting point. Based on this principle, Tan et al. developed a novel one‐pot‐RPA‐CRISPR/Cas12a assay where RPA and CRISPR/Cas12a systems were separated by paraffin for visual, sensitive, and specific detection of six common respiratory bacteria with a LOD of 2.5 copies µL^−1^ plasmids (Figure 4A).^[^ ^41^ ^]^ Similarly, paraffin capsules encapsulating freeze‐dried CRISPR reagents effectively achieved separation from INA systems.^[^ ^85^, ^86^ ^]^

Although spatial isolation avoids contamination risks, extra operations such as centrifugation and heating are still required, which limits the suitability of POCT. In addition, aerosol contamination from INA may cause premature activation of CRISPR/Cas, and CRISPR solutions on the tube cap may be spilled if a substantial amount is present.^[^ ^62^, ^87^ ^]^ To address these issues, Fu et al. used a round cap to temporarily store more CRISPR/Cas solution than a flat cap, but this issue has not yet been fully resolved.^[^ ^88^ ^]^ Besides, the amplification reagents were sealed at the bottom of the tube with mineral oil to reduce aerosol contamination, but it still needed cumbersome operations.^[^ ^89^ ^]^ Xu et al. constructed an integrated tube, mainly made of polydimethylsiloxane (PDMS)/glass capillary and PCR tube for visually detecting SARS‐CoV‐2 with a one‐pot assay. As reported, the Cas12a reagent sealed in the PDMS/glass capillary tube was dropped into the amplified products for CRISPR detection by gently pressing the rubber tip after a 15‐minute RT‐RPA reaction.^[^ ^87^ ^]^ Utilizing this approach, the interaction between Cas12a and the amplification products can be performed efficiently by simple manual procedures, so that no additional instruments are required.

#### 3.1.2 Phase Separation

To simplify spatial separation strategies, additives like sucrose,^[^ ^73^ ^]^ glycerol,^[^ ^74^, ^90^ ^]^ and agarose^[^ ^48^ ^]^ are used to influence the viscosity or density of the solution for a phase‐separating manner. These additives, combined with either the CRISPR or amplification, hinder the migration of biomolecules and reduce the inhibitory effects of CRISPR on amplification. As diffusion proceeds, the sufficient amplicon generated will be dynamically mixed with the CRISPR/Cas system and trigger its cleavage activity. For example, a dynamic aqueous multiphase reaction (DAMR) system utilizing sucrose to form a density difference was established for a simple, rapid, sensitive, and quantitative one‐pot assay. This system and a 3D‐printed microfluidic device were utilized to quantitatively detect HPV 16 and 18 DNAs with LOD of 10 and 100 copies per reaction within one hour (Figure 4B).^[^ ^73^ ^]^ The DAMR system also was combined with a microfluidic chip for simultaneous malaria infection screening and *Plasmodium* species genotyping.^[^ ^91^ ^]^ While the phase separation strategy avoids additional operation, the implementation of it requires careful operation, which can introduce reproducibility issues and then affect the stability of the results. Moreover, the reliance on slow molecular diffusion across viscous phases inherent to this strategy can delay signal generation, thereby prolonging detection time. These factors not only limit its suitability for POCT but also introduce complexities in reaction kinetics, posing challenges for standardization and large‐scale application.

#### 3.1.3 Isolation in Time Dimension

Time‐based isolation strategies enable CRISPR activation only after adequate amplicon accumulation, preventing premature interference with the amplification process. Photocontrolled technologies^[^ ^24^ ^]^ and crRNA modifications^[^ ^75^ ^]^ are used to achieve this. As a noninvasive, rapid, high‐spatial‐resolution biochemical reaction switch, the photoactivation strategy has long been used as a time controller for gene editing in vivo to prevent off‐target reactions.^[^ ^92^, ^93^, ^94^ ^]^ To ensure sufficient accumulation of amplicon before the CRISPR reaction, photocontrolled technology was further applied in one‐pot CRISPR‐based detection (Figure 4C).

Zhou's team demonstrated that the photocontrolled CRISPR significantly improved detection sensitivity, achieving a more than two‐order magnitude increase compared to the one‐pot assay directly mixing two systems.^[^ ^24^ ^]^ In particular, the crRNA was blocked by protective oligonucleotides (p‐oligo) containing a PC linker to silence the cleavage of CRISPR/Cas12a, which could be restored by the light‐controlled activation. Hence, in the one‐pot detection system, the silent CRISPR/Cas12a system did not interfere with INA, which ensured sufficient accumulation of amplicon. The CRISPR/Cas12a system could be activated rapidly (a few seconds) by light irradiation after amplification was fully completed, which achieved separation of RPA and CRISPR/Cas12a detection in a temporal dimension. In addition, the team further developed a new light‐start CRISPR/Cas12 system using photochemically activated caged crRNA that prevented the Watson–Crick base pairing between crRNA and the target to silence the CRISPR/Cas12 activity.^[^ ^42^ ^]^ Similarly, Chen et al. employed PC linker to develop a photocontrolled one‐pot assay, achieving a LOD of 2.5 copies reaction^−1^ within 40 min.^[^ ^94^ ^]^

Although the above approaches avoid aerosol contamination and system incompatibility, they still have the disadvantage of low universality because the modification position and number of PC linkers or NPOM require cumbersome trial‐and‐error screening according to the target sequence. In addition, the yield of chemically modified crRNA and caging efficiency bring limitations to the above strategies.^[^ ^95^ ^]^ Therefore, Liu et al. proposed a universal and accessible acylation strategy that was independent of the crRNA sequences to regulate the CRISPR/Cas12a system by efficient acylation of 2′‐hydroxyls on the crRNA strand with photolabile agents (PLGs). The introduction of PLGs enabled efficient suppression of crRNA function and rapid recovery of CRISPR/Cas12a reaction upon brief exposure to light. Based on this strategy, they constructed a universal photo‐initiated CRISPR/Cas12a system for a robust one‐pot testing (POIROT) platform integrated with RPA, which was two orders of magnitude more sensitive than the one‐pot assay directly mixing two systems and comparable to the two‐step assay.^[^ ^66^ ^]^ This strategy undoubtedly makes the photocontrolled CRISPR more universal, convenient, and idealized. Despite these advantages, photoactivation‐based strategies frequently require chemical modification, which may involve complex reactions and tedious operations, increasing additional synthesis costs and manual labor.

Apart from the photoactivation strategy, temporal separation can be facilitated by designing silenced crRNAs to block the CRISPR reaction, in which the function of crRNA is restored through enzyme cleavage, nucleic acid strand substitution, or cascade reactions.^[^ ^75^, ^96^, ^97^ ^]^ A one‐pot detection of microRNAs (miRNAs) was developed via integrating an RNA‐based CHA circuit with CRISPR/Cas12a.^[^ ^75^ ^]^ In this system, the crRNA sequence was partially blocked to prevent interacting with Cas12a. The CHA circuit would be triggered in the presence of target miRNA to turn the blocked crRNAs into pre‐crRNAs, which regained their function after being processed into mature crRNAs by Cas12a. Subsequently, Cas12a‐crRNA complexes activated its DNase activity to cleave the reporters for signal output. Analogously, circular‐crRNA and enzyme‐responsive steric hindrance‐based branched inhibitors also were designed to silence crRNA. The former was activated by the cleavage of DNA enzyme generated by target triggering,^[^ ^96^ ^]^ while the latter regained function through the recognition and incision of APE1 with the presence of the target.^[^ ^97^ ^]^

In addition, the nicked PAM has been explored to delay CRISPR activation and achieve temporal separation of the amplification and detection processes. Wang et al. reported an antibody‐aptamer sandwich‐like immunosensor that combined RCA with a nicked‐PAM CRISPR/Cas12a system for the detection of C‐reactive protein (CRP).^[^ ^98^ ^]^ In this system, RCA reaction guided by aptamer‐primer probe occurred subsequent to the capture of the CRP, synthesizing long ssDNA. The NTS sequence was designed to be split at the PAM site into two fragments (NTS‐a and NTS‐b). TS and NTS‐a bound to long ssDNA to form a double‐stranded complex consisting of the TS, NTS‐a, and NTS‐b. This fully formed PAM‐containing duplex subsequently activated the CRISPR/Cas12a system, generating a fluorescence signal for detection.

On the basis of the above strategies, some accessories have been introduced to achieve more precise regulation of CRISPR activity, such as complementary strands to crRNA,^[^ ^27^ ^]^ overhanging activators,^[^ ^99^ ^]^ and G‐quadruplex.^[^ ^100^ ^]^ Some of these methods have already been implemented for enhanced performance of one‐pot detection. For example, external RNA accessories (ERAs) with different structures, lengths and mismatches could be designed as protectors to bind to part of the spacer region of crRNA to form a toehold‐containing double‐stranded structure that allowed toehold‐mediated strand displacement reaction determining the pairwise binding of the activator to crRNA, thereby realizing precise control of the activation level of Cas (Figure 4C).^[^ ^27^ ^]^ Besides, recent research displayed precise modulation of Cas12a activity via an RNA G‐quadruplex (RG4)‐modified crRNA and RG4 stabilizers, which was introduced further into a one‐pot assay to realize temporal separation.^[^ ^100^ ^]^ Although some strategies for regulating CRISPR activity have not yet been applied to construct a one‐pot assay, the value of providing promising technical references is undeniable.

### 3.2 Optimization of Components

#### 3.2.1 Modification of crRNA

A key factor in the incompatibility between CRISPR and INA is the presence of competitive reactions. Therefore, the balance of reaction kinetics is a crucial breakthrough point for constructing one‐pot detection with superior performance. Michaelis–Menten model^[^ ^101^ ^]^ for trans‐cleavage activity of Cas12a was established and validated, which described the evolution of the product (reporter cleavage) versus time using the following scaling equation:

(1) $$\frac{d\left[P\right]}{dt}\approx \frac{k_{\mathrm{cat}}E_{0}}{K_{M}}\left(S_{0}-\left[P\right]\right)$$

The equation was used to further derive an expression for the scaled concentration of the product formed versus time as:

(2) $$\frac{\left[P\right]\left(t\right)}{S_{0}}\approx \left(1-\exp\left(-\frac{t}{\tau }\right)\right)$$

where τ  =  *K_M_*/*k_cat_E*_0_. τ refers to a time scale determining the characteristic time for completing trans‐cleavage, while [P](t)/S_0_ represents the fraction of cleaved reporters relative to the total initial reporters. The equations show that τ is proportional to *K_M_* and inversely proportional to *k_cat_*.^[^ ^102^ ^]^ Besides, the factors influencing the rate constant *k_cat_*/*K_M_* of enzymatic reactions include the Cas type, crRNA, incubation time, pH, and temperature.^[^ ^103^ ^]^ Additionally, the structure of crRNA affects the binding affinity of Cas12a with target DNA, influencing Cas12a activity.^[^ ^104^ ^]^ Therefore, suboptimal crRNA provides a promising approach to ensure that amplification dominates the pre‐reaction period.

**Figure 5:**
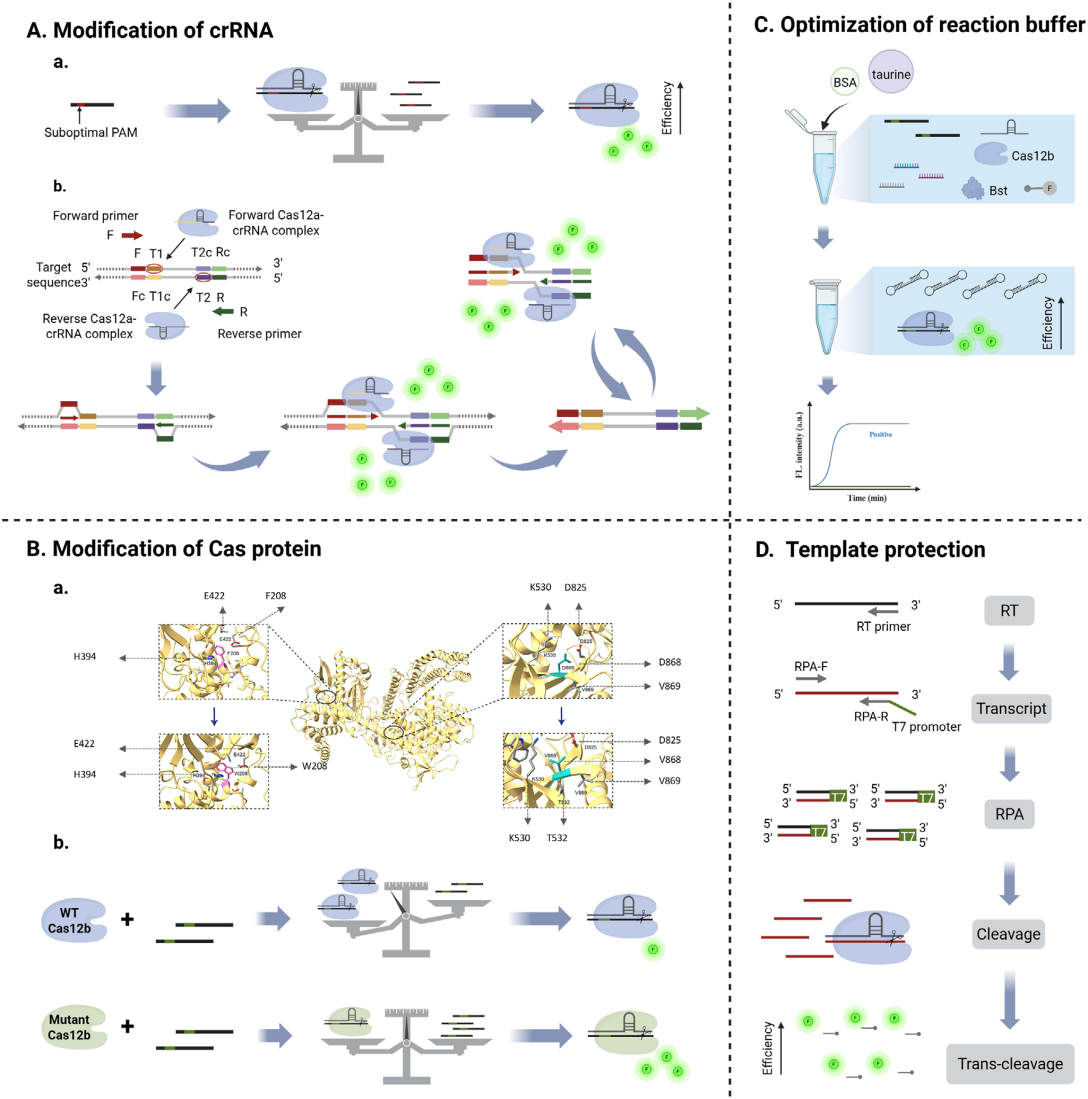
Schematic of optimization of the component. A) Modification of crRNA. a) Utilizing suboptimal PAM, cleavage kinetics were tuned for balancing amplification and CRISPR to improve sensitivity.^[^ ^64^ ^]^ b) AIOD‐CRISPR introduced dual PAM‐free crRNAs to restrain the cleavage of the templates for generating enough amplicons.^[^ ^65^ ^]^ B) Modification of Cas protein. a) Mutations were introduced into the wild‐type BrCas12b to improve thermostability. Reproduced with permission.^[^ ^115^ ^]^ 2025, Elsevier. b) PAM‐interacting domains of Cas were modified to establish a balance between amplification and cleavage of Cas to improve sensitivity.^[^ ^40^ ^]^ C) Optimization of reaction buffer. BSA and taurine were added to the one‐pot system to directly improve detection sensitivity.^[^ ^76^ ^]^ D) Template protection. Amplicons complementary to the templates were obtained by coupling the T7 promoter to the reverse primer for protecting the templates from cleavage.^[^ ^40^ ^]^

In the CRISPR/Cas system, a specific sequence adjacent to the target site, called PAM, is usually required to initiate the target recognition.^[^ ^105^ ^]^ LbCas12a and AsCas12a typically recognize the TTTV PAM (canonical PAM), but can also recognize non‐canonical PAMs containing C with reduced efficiency, such as CTTV, TCTV, and TTCV.^[^ ^46^, ^106^, ^107^ ^]^ However, excessive Cas activation by crRNA with canonical PAMs can lead to degradation of INA templates in one‐pot detection, compromising amplification efficiency.^[^ ^77^ ^]^ Previous studies indicated that the detection sensitivity and reproducibility of the one‐pot assay could be significantly improved by using suboptimal PAMs and modified crRNA sequences to adjust cleavage kinetics.^[^ ^40^, ^64^ ^]^ In 2022, Lu et al. proposed that the kinetics of Cas12a‐mediated trans‐cleavage of fluorescent DNA reporters and cis‐cleavage of substrates could be slowed down by using suboptimal PAM, which allowed the accumulation of amplicons (**Figure** **5**A). In this study, amplicon generation in the one‐pot reaction was monitored to assess the interaction between CRISPR and INA. Results showed that amplicons were detectable as early as 2 min when a suboptimal PAM was used, whereas detection was delayed to 8–10 min when a canonical PAM was employed. These findings indicate that CRISPR activity can inhibit INA, and that the use of suboptimal PAM sequences helps to balance the amplification and cleavage reactions within the one‐pot system. Based on this principle, they developed a rapid sPAMC platform with comparable speed as isothermal amplification alone.^[^ ^64^ ^]^ This approach has since enabled one‐pot detection of pathogens such as monkeypox virus (MPXV),^[^ ^108^ ^]^ *Echinococcus granulosus*,^[^ ^109^ ^]^ SARS‐CoV‐2, ASFV, HPV16, and HPV18.^[^ ^110^ ^]^

The crRNAs that are not restricted by the PAM sequence are also designed to increase the efficiency of one‐pot detection. Ding et al. developed an All‐In‐One Dual CRISPR‐Cas12a (AIOD‐CRISPR) assay, which utilized dual crRNAs without PAM sequence to enable sensitive and robust SARS‐CoV‐2 detection in one pot (Figure 5A).^[^ ^65^ ^]^ Similarly, Lin et al. developed a one‐pot RPA‐CRISPR detection system for rapid and visual detection, which was unrestricted by the PAM sequence and could detect 0.2 copies µL^−1^ of DNA and 0.4 copies µL^−1^ of RNA. They found that the trans‐cleavage activity of Cas12a was unaffected by PAM status, while non‐PAM crRNAs showed weaker cis‐cleavage, reducing template loss and facilitating one‐pot detection.^[^ ^111^ ^]^

In addition to the design of PAM sequences, other means have also been adopted to modify crRNA for reducing the cis‐cleavage activity of CRISPR. The ssDNA‐modified crRNA, consisting of wild‐type crRNA (Wt‐crRNA) and ssDNA extensions at the 3′ and 5′ ends, was employed for one‐pot diagnostic, exhibiting an almost 1000‐fold increase in sensitivity compared to the one‐pot assay using Wt‐crRNA.^[^ ^112^ ^]^ While suboptimal PAMs enable one‐pot detection, they typically balance reaction kinetics at the expense of reducing CRISPR activity. To achieve reaction balance for maximum detection efficiency, it is usually necessary to evaluate and screen the types and sites of suboptimal PAM for a specific target, which limits its universality and increases workload. Similarly, strategy using suboptimal crRNAs also needs to be optimized for specific targets, introducing the same limitations.

#### 3.2.2 Modification of Cas Protein

Compatible reaction conditions are essential for effective one‐pot detection combining INA and CRISPR. However, most CRISPR reactions with Cas proteins from mesophilic bacteria run at ≈37 °C, whereas INA works optimally at different temperatures such as 40 or 65 °C. The protein engineering of Cas enzymes enhances one‐pot detection by improving thermostability to address temperature mismatches and adjusting reaction kinetics to balance amplification and cleavage.

To date, a series of thermostable Cas enzymes have been identified and applied in CRISPR detection. A primary strategy in the development of thermostable Cas enzymes involves the exploration and isolation of natural Cas proteins exhibiting diverse characteristics from various species. For example, BrCas12b from *Brevibacillus sp*. showed high *trans*‐cleavage up to 64 °C without the need for supplemental additives, which was applied to establish one‐pot detection called CRISPR‐SPADE for detection of SARS‐CoV‐2 variants of concern (VOCs).^[^ ^67^ ^]^ Two novel Cas12a proteins, exhibiting functional activity at elevated temperatures beyond those of commonly utilized orthologs, were identified and characterized.^[^ ^113^ ^]^ Later, a thermostable ortholog of the Cas13a family from the thermophilic organism *Thermoclostridium caenicola* (TccCas13a) was identified, possessing robust cis‐ and trans‐activities at a broad temperature range from 37 to 70 °C.^[^ ^56^ ^]^ Benefiting from the thermostability, TccCas13a was used to develop a sensitive, robust, and rapid one‐pot assay (OPTIMA‐dx) for the detection of SARS‐CoV‐2. However, the cumbersome screening of species and the inherent limitations in the quantity of natural proteins may retard the development of thermostable Cas enzymes.

Another important method to improve thermostability is the modification of the Cas enzyme using protein engineering, bringing more potentiality. The previous research revealed that the thermostability of enzymes was mainly related to their secondary and tertiary structures.^[^ ^114^ ^]^ Therefore, Nguyen et al. developed an engineered Cas12b with improved thermostability by introducing mutations to wild‐type BrCas12b to tighten its hydrophobic cores, which was utilized to establish a one‐pot detection assay called SPLENDID (Figure 5B).^[^ ^115^ ^]^ They first utilized AlphaFold and SWISS‐MODEL to predict the structure of BrCas12b, then carried out sequence alignment of the models with the most homologous solved Cas12b ortholog to identify functional domains for thermostability and mutation sites of amino acid, among which hydrophobic substitutions in certain regions were stable. Subsequently, predictors of thermostability in terms of hydrophobicity, compactness, hydrogen bonding, and salt bridges were analyzed to select the best candidates.

In addition, researchers have adapted the reaction kinetics to balance amplification and cleavage by protein engineering of Cas enzymes, which is a commendable solution to the problem that suboptimal PAMs effectively deal with Cas12a but are unsuitable for Cas12b and Cas13 featuring unstrict or no requirements of PAM.^[^ ^40^ ^]^ A universal method was identified through protein engineering of PAM‐interacting domains to enhance the performance of one‐pot testing for Cas12 orthologs (Figure 5B). Based on this strategy, the engineered Cas12b mutant demonstrated the capability to detect as low as 0.5 copies µL^−1^ SARS‐CoV‐2 within 15 min.^[^ ^40^ ^]^ In addition, the team recently developed a mutant Cas protein with reduced cis‐cleavage via residue substitution in the interacting domain with PAM, demonstrating excellent one‐pot detection performance.^[^ ^116^ ^]^

Modification of Cas proteins based on protein engineering provides an effective approach to improve the sensitivity of one‐pot detection via developing thermostable Cas and adjusting the reaction kinetics. However, this strategy requires complex characterization, screening, and engineering of Cas.

#### 3.2.3 Optimization of Reaction Buffer

The reaction buffer plays a critical role in providing optimal biochemical conditions, including appropriate pH and ion concentrations to support the activity of Cas proteins. The Cas effectors currently used in nucleic acid detection, including Cas3, Cas9, Cas10, Cas12, Cas13, and Cas14, typically exhibit optimal activity at pH values ranging from 7 to 8. This pH range can be fine‐tuned according to the specific requirements of the detection assay to maximize performance. The core buffer compositions required for these Cas proteins are generally similar, with Mg^2+^ being an essential cofactor for their catalytic activity. In addition, components such as glycerol, bovine serum albumin (BSA), dithiothreitol, and CoCl_2_ may be incorporated as needed to enhance enzyme stability, promote proper folding, or improve overall detection performance.^[^ ^3^, ^5^, ^117^, ^118^, ^119^, ^120^, ^121^, ^122^ ^]^ Therefore, optimizing the reaction buffer is another critical factor in improving the efficiency and sensitivity of one‐pot detection, but the screening of each condition one by one is complex and time‐consuming.

Statistical design of experiments^[^ ^123^ ^]^ and mathematical models^[^ ^124^, ^125^ ^]^ provide efficient tools for the modifications including adjusting component ratios, concentrations, and temperatures. Among them, chemical additives can directly improve reaction performance. Chemical additives such as betaine,^[^ ^126^, ^127^, ^128^, ^129^, ^130^ ^]^ glycerol,^[^ ^130^ ^]^ dithiothreitol,^[^ ^128^ ^]^ and dimethyl sulfoxide (DMSO)^[^ ^128^, ^129^, ^130^, ^131^ ^]^ have been proved that they can improve the efficiency and specificity of PCR or INA. In CRISPR systems, additives such as BSA and L‐proline have been demonstrated to enhance the activity of Cas12a and Cas13a enzymes while concurrently conferring protection against thermal denaturation.^[^ ^132^ ^]^ Recent studies have expanded the types of additives for one‐pot CRISPR/Cas system, demonstrating the efficacy of glycerol, BSA,^[^ ^133^ ^]^ taurine, glycine,^[^ ^76^, ^134^ ^]^ trehalose,^[^ ^135^ ^]^ and betaine^[^ ^136^ ^]^ in enhancing detection sensitivity. In a study of the one‐pot detection method of MPXV, researchers investigated the effects of chemical additives with different concentrations (including L‐proline, urea, betaine, glycerol, DMSO, and BSA) on Cas12a cleavage, among which 5% glycerol and BSA significantly increased sensitivity by two orders of magnitude compared with the additive‐free system.^[^ ^133^ ^]^ Xu et al. also confirmed that BSA and taurine could improve the efficiency of one‐pot detection (Figure 5C), and also found that glycine had a similar effect.^[^ ^76^ ^]^ In one‐pot RPA‐CRISPR/Cas12a system for detection of the *mecA* gene, the sensitivity was increased tenfold by adding glycerol and betaine, achieving 10 copies µL^−1^.^[^ ^136^ ^]^ Besides, there is also a one‐pot detection system enhanced by synergizing chemical additives with other one‐pot strategies. A DMSO‐enhanced one‐pot HDA‐CRISPR/Cas12a biosensor for detection of MPXV was proposed, consisting of DMSO‐enhanced HDA at the bottom and Cas12a reagents pre‐added to the caps.^[^ ^137^ ^]^

In addition, divalent cations are capable of promoting pre‐crRNA processing by Cas12a in the activation^[^ ^138^ ^]^ and DNA cleavage steps.^[^ ^139^ ^]^ Feng Zhang's team studied the effect of ions on the collateral cleavage of Cas13 orthologs, suggesting that Ca^2+^, Mg^2+^, Mn^2+^, and Ni^2+^ offered better collateral cleavage.^[^ ^140^ ^]^ Therefore, there is a possibility that ions can improve the sensitivity of the one‐pot system.

Interestingly, several additives with inhibitory effects on CRISPR were applied to regulate CRISPR activity to promote the accumulation of amplicons for indirectly enhancing one‐pot detection. For instance, a universal one‐pot assay was established, where heparin sodium interfering with the Cas12a‐crRNA binding process was added to precisely tune the cis‐cleavage of CRISPR.^[^ ^141^ ^]^

#### 3.2.4 Template Protection

CRISPR/Cas13‐based RNA detection often involves reverse transcription and preamplification, such as SHERLOCK^[^ ^3^ ^]^ and SHINE.^[^ ^50^ ^]^ However, Cas13a‐mediated cleavage can interfere with these processes in one‐pot setups. To mitigate this, researchers have employed strategies to protect amplification templates while maintaining CRISPR activity.

For example, the SARS‐CoV‐2‐derived sequences in the RPA primers and crRNA were replaced with their reverse‐complementary counterparts, avoiding the competition between CRISPR and RT‐RPA, and improving the sensitivity and speed of one‐pot SHERLOCK at a low target concentration.^[^ ^142^ ^]^ Tong et al. also reported a novel one‐pot detection of SARS‐CoV‐2, in which cDNA obtained by reverse transcription from the viral RNA was amplified using a reverse primer incorporating a T7 promoter sequence, thereby ensuring that the RNA transcribed from the DNA amplicons was reverse‐complementary to the viral RNA. Therefore, Cas13a‐crRNA designed to specifically cleave RNA amplicons would not damage the template RNA (Figure 5D).^[^ ^40^ ^]^ Similarly, a one‐pot NASBA‐Cas13a detection assay was established, where target RNAs were first converted and amplified into antisense activator RNAs through NASBA. These activator RNAs subsequently initiated CRISPR/Cas13a activity, while the design effectively prevented degradation of the amplification templates.^[^ ^125^ ^]^ Moreover, a platform for extracellular vesicle (EV) messenger RNA detection, called Self‐amplified and CRISPR‐aided Operation to Profile EVs (SCOPE), was reported.^[^ ^143^ ^]^ In this system, a signal template is introduced that contains an RNA segment labeled with a quenched fluorescent dye and a DNA sequence serving as a template for T7 RNA polymerase. The RNA segment functions as the substrate for CRISPR/Cas13a trans‐cleavage, whereas the DNA template drives T7 transcription, enabling built‐in dual amplification and providing protection for the amplification template.

In addition, a novel PAM‐free one‐pot asymmetric RPA (aRPA) coupled with a CRISPR/Cas12b assay (OAR‐CRISPR) was established.^[^ ^77^ ^]^ In the platform, dsDNA was generated by RPA with sufficient forward and reverse primers at an early stage. After the depletion of reverse primers, PAM‐free aRPA initiated the synthesis of ssDNA based on the dsDNA templates with forward primers alone, and PAM‐free crRNA was designed to recognize ssDNA amplicons. The requirement for PAM sequences in the cleavage of dsDNA effectively avoided cleaving the amplification templates. Cao et al. reported a CRISPR‐based one‐pot LAMP (CoLAMP) assay for POCT of SARS‐CoV‐2, in which AapCas12b crRNA was designed to recognize the activator sequence that sited in the loop region of the LAMP product instead of the target sequences, so that the target could not be digested before amplification.^[^ ^144^ ^]^

The approach that the targets serve as templates of INA for exponential generation of activators with different sequences from the templates to initiate the CRISPR reaction is also an effective strategy to protect amplification templates. For example, SDA based on three‐way junction architecture was combined with CRISPR/Cas12a to develop a one‐pot detection platform of H5N1, where three hairpins were introduced to identify the target and form a three‐way junction acting as primer‐template junctions to drive SDA for exponential production of activators initiating CRISPR.^[^ ^145^ ^]^ Similarly, a one‐pot SDA‐CRISPR‐based biosensor was constructed for sensitive detection of 8‐oxoguanine DNA glycosylases with the LOD of 4.24 × 10^−9^ U µL^−1^.^[^ ^146^ ^]^ The biosensor involved a hairpin probe, which could be processed by 8‐oxoguanine DNA glycosylase and polynucleotide kinase to initiate the quadratic SDA for producing large amounts of activators, thereby activating CRISPR/Cas12a.

In summary, this strategy of template protection to reduce the inhibition of amplification is mainly accomplished by altering the recognition sequence of crRNA. Whereas, some methods may involve complex optimization of primers, limiting the universal application.

### 3.3 Development of Integrated Devices

#### 3.3.1 Tube‐In‐Tube Vessel

To overcome the limitations of physical isolation methods that CRISPR reagents are temporarily stored in the tube cap, researchers have developed “tube‐in‐tube” vessels as an innovative one‐pot testing platform.^[^ ^62^ ^]^ This vessel consists of an inner and an outer tube. The inner tube, in which the CRISPR reagents are stored, has hydrophobic pores at its bottom. Once amplification in the outer tube is completed, these pores enable the reagents to be transferred to the outer tube by centrifugation or vibration for the subsequent cleavage reaction.

**Figure 6:**
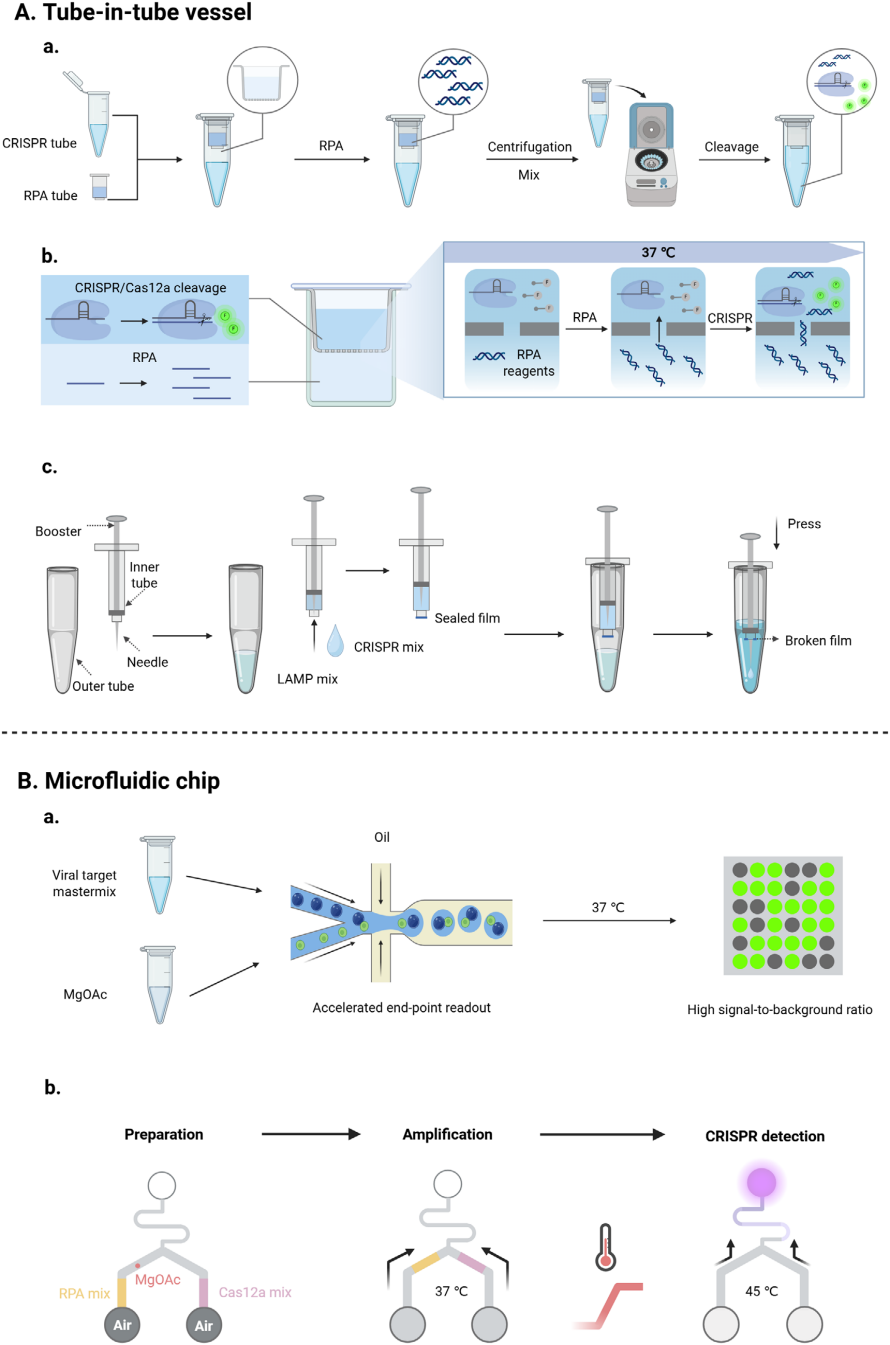
Schematic of the development of integrated devices. A) Tube‐in‐tube vessel. a) RPA and CRISPR reagents were physically separated by inner and outer tubes, and the two systems were mixed by centrifugation for cleavage after amplification.^[^ ^37^ ^]^ b) STAR used capillary channels between inner and outer tubes for physical separation and connection of the two systems.^[^ ^78^ ^]^ c) Amplicons were mixed with CRISPR reagents by piercing the seal at the bottom of the inner tube with a needle after amplification.^[^ ^62^ ^]^ B) Microfluidic chip. a) A Y‐shaped microfluidic device with two inlets for MgOAc and target‐included master mix to avoid the early start of the RPA reaction was used to establish MEDICA for quantification.^[^ ^79^ ^]^ b) Thermometer‐inspired microfluidic biosensing platform seamlessly integrated CRISPR and RPA through temperature‐programmed air expansion.^[^ ^149^ ^]^

For example, Hu et al. combined CRISPR/Cas13a and RPA in a disposable tube‐in‐tube vessel for the one‐pot detection of ASFV and SARS‐CoV‐2 (**Figure** **6**A).^[^ ^37^ ^]^ To facilitate operations, Han et al.^[^ ^62^ ^]^ sealed the bottom of the inner tube with a film, which was punctured by a needle fixed on the booster to allow the transfer of the CRISPR reagents after amplification (Figure 6A). Similarly, the device with a two‐layer structure integrated INA and CRISPR in a closed container, allowing the amplification products to enter the CRISPR reaction chamber by removing the limit ring of the reaction unit and pressing vertically.^[^ ^147^ ^]^ Additionally, capillary channels were employed in some designs to serve as a semi‐barrier, facilitating the separation and connection of the systems in inner and outer tubes, eliminating the extra operation (Figure 6A).^[^ ^78^ ^]^ The tube‐in‐tube vessel effectively addresses issues such as complex operation, limited reagent storage in the tube lid, and premature CRISPR activation due to aerosolized amplicons. However, the strategy is hindered by the need for intricate vessel design, extended reaction times, and increased costs, which pose challenges for widespread adoption.

#### 3.3.2 Microfluidic Chip

To deal with the incompatibility between the INA and CRISPR system, as well as the difficulty of CRISPR‐based multiplex detection, microfluidic technology was utilized to realize one‐pot detection. Microfluidic chips integrate amplification, CRISPR‐based detection, and sample pretreatment steps (e.g., nucleic acid extraction) into a single platform, which offers several advantages including portability, high throughput, low reagent consumption, and good controllability. Reaction solutions can be transported to microchambers by pumps, centrifugal force, or capillary force, enabling automated workflows. Notably, capillary force‐driven systems operate spontaneously without the need for additional instruments. Typically, INA is performed in a designated microchamber, and the amplification products are transferred to another chamber for the CRISPR reaction by centrifugation or other methods.

For example, an automated, portable microfluidic detection system was developed that integrated rotary valve‐assisted sample pretreatment and RPA‐T7‐Cas13a into a one‐pot detection system, which was validated by detecting *Group B streptococci* DNA.^[^ ^148^ ^]^ Another fully enclosed, highly integrated microfluidic system (LOC‐CRISPR) was established to extract viral nucleic acids, perform RPA, and carry out CRISPR‐based detection, enabling the identification of multiple respiratory viruses and their variants.^[^ ^28^ ^]^ Besides, a microfluidic platform seamlessly integrated CRISPR and RPA through temperature‐programmed air expansion to develop a one‐pot detection for syphilis (Figure 6B).^[^ ^149^ ^]^ Despite of innovativeness of this thermometer‐inspired microfluidic biosensing platform, additional instruments required for temperature control limit its application for POCT.

As a specialized microfluidic chip, lab‐on‐a‐disc (LOAD) platforms are widely studied due to the advantages that highly efficient fluid transportation only requires a compact motor to realize centrifugal pumping and is not strongly dependent on the physicochemical properties of the fluid.^[^ ^150^, ^151^ ^]^ Unlike many microfluidic systems, LOAD permits fully sealed operation without air venting or specialized loading procedures, significantly reducing the risk of contamination.^[^ ^152^ ^]^ Due to these design strengths, LOAD has been widely adopted for sample preparation and molecular detection workflows.^[^ ^151^ ^]^ Researchers have been successfully integrated sample pretreatment, INA, and CRISPR detection into compact LOAD systems. A notable example is a portable centrifugal microfluidic testing (POCMT) system, which integrates magnetic beads‐based nucleic acid extraction, RAA, and the CRISPR/Cas13a system into a single LOAD device. This platform provides precise fluid control, thermal cycling, and real‐time readout via a handheld instrument, demonstrating efficient, automated, one‐pot molecular diagnostics.^[^ ^153^ ^]^

In addition, the combination of droplet microfluidic chip and digital CRISPR/Cas detection has the potential to realize rapid quantitative detection. Nucleic acids are dispersed in thousands of microreactors through a microfluidic chip for amplification, which increases the local concentration of reaction components, facilitating rapid amplification. The CRISPR/Cas system then generates fluorescence signals, allowing absolute quantification of nucleic acid molecules by counting the positive units.^[^ ^154^ ^]^ The first digital CRISPR/Cas‐assisted assay, called coined digitization‐enhanced CRISPR/Cas‐assisted one‐pot virus detection (deCOViD), was developed by Park et al. for SARS‐CoV‐2 detection.^[^ ^155^ ^]^ Similarly, Liu et al.^[^ ^79^ ^]^ designed a Y‐shaped microfluidic device with dual inlets for MgOAc and the target master mix, effectively preventing premature initiation of RPA and achieving endpoint quantification within 25 min (Figure 6B). Microfluidic technology, with a high level of integration, automation, and low sample requirements, is a promising approach for POCT. However, the complexity of device design, particularly reversible valve‐assisted chips, and the associated economic costs limit its broader application in resource‐limited settings.

The commercialization of integrated devices will promote the extensive application of one‐pot CRISPR‐based detection platforms in clinical diagnosis, but most of the integrated devices mentioned above are still in the research stage.^[^ ^37^, ^62^, ^78^ ^]^ Encouragingly, some similar devices that can assist with one‐pot detection have been successfully commercialized.^[^ ^156^, ^157^, ^158^, ^159^, ^160^ ^]^ To present the translational potential and operational feasibility of each device, the devices related to one‐pot CRISPR‐based detection are summarized according to their advantages, disadvantages, and commercialization (**Table** **2**).

**Table 2:** Comparison of integrated devices.

Device	Commercialization	advantages	disadvantages	Refs.
Tube‐in‐tube vessel with hydrophobic pores	No	Low cost	Requirement for size optimization, 3D printing	[37]
Tube‐in‐tube vessel with needle	No	Low cost, simple manual operation	Complex fabrication	[62]
Tube‐in‐tube vessel with limit ring	Yes	Simple manual operation	Higher cost	[147, 156]
Tube‐in‐tube vessel with capillary channels	No	Low cost, automation	Requirement for size optimization, 3D printing	[78]
Lab‐on‐chip	Yes	Highly integrated, simple manual operation, portable, feasibility of multiplex detection	Higher cost	[157]
LOAD	Yes		Higher cost, requirement for centrifugation	[157]
The BG‐Nova‐X8 Integrated Rapid Nucleic Acid Detection System	Yes	Fully automatic, high‐throughput	High cost, large scale	[158]
SynsorPocket‐One	Yes	Handheld, automatic	Higher cost	[159]
Lab in Tube	Yes	Portable	Requirement for the fluorescence signal reader	[160]

## 4 Conclusion and Future Perspectives

The CRISPR system has been proven as a revolutionary technique for nucleic acid detection, offering sensitivity, specificity, and simplicity in operation. However, its practical application in real‐world diagnostic scenarios requires coupling with INA to achieve the required sensitivity. Despite considerable advantages, several challenges remain, including aerosol contamination and operational complexity due to multi‐step workflows. These limitations hinder the widespread adoption of CRISPR‐based detection technologies. To address these challenges, the development of robust one‐pot INA‐CRISPR detection systems is crucial. However, achieving a truly effective one‐pot system is a major challenge due to inherent incompatibilities between the CRISPR and INA, such as premature cleavage of templates, primer degradation, inhibitory effect of reagents, and reaction temperature mismatches.

Currently, significant progress has been made in developing effective one‐pot CRISPR‐based detection methods by employing strategies such as system isolation, component optimization, and device integration. Compared to traditional clinical diagnostics, these one‐pot CRISPR assays have significant advantages in speed, operational simplicity, and cost‑effectiveness. For example, the bacterial culture methods typically necessitate 3 to 5 days to confirm infections, while faster assays including enzyme‐linked immunosorbent assay and PCR still often take up to a full day.^[^ ^161^ ^]^ By contrast, one‐pot CRISPR diagnostics can generally complete the detection within one hour, drastically reducing turnaround time. Moreover, traditional approaches rely on expensive instruments and central laboratories for bacterial culture and thermal cycling programs, but one‐pot CRISPR‐based detection only requires constant temperature heating equipment and blue light, making them ideally suited for resource‑limited or point‑of‑care settings.^[^ ^162^ ^]^

However, as an emerging technology, the commercialization of CRISPR detection platforms is still under continuous exploration, especially the one‐pot assays. As far as we know, a batch of one‐pot CRISPR detection methods, including STOPCovid and NuRapid, are currently in the clinical validation stage.^[^ ^156^, ^163^ ^]^ STOPCovid is a one‐pot assay for SARS‐CoV‐2 detection based on SHERLOCK with a LOD of 100 copies reaction^−1^ for nasopharyngeal swabs within 45 min, in which INA and CRISPR systems are directly mixed and optimized, with a cost of approximately US $1 per reaction.^[^ ^164^ ^]^ However, STOPCovid still requires a separate nucleic acid extraction step. NuRapid is an extraction‐free nucleic acid detection platform integrating INA and CRISPR using a tube‐in‐tube device, which has been applied to the diagnosis of COVID‐19 and tuberculosis.^[^ ^156^ ^]^ NuRapid is able to detect as low as 1–3 copies per reaction within 30 min, only requiring one step for sample transfer. The cost of NuRapid only involves reagents, disposable consumables, and reusable portable temperature controllers.

**Figure 7:**
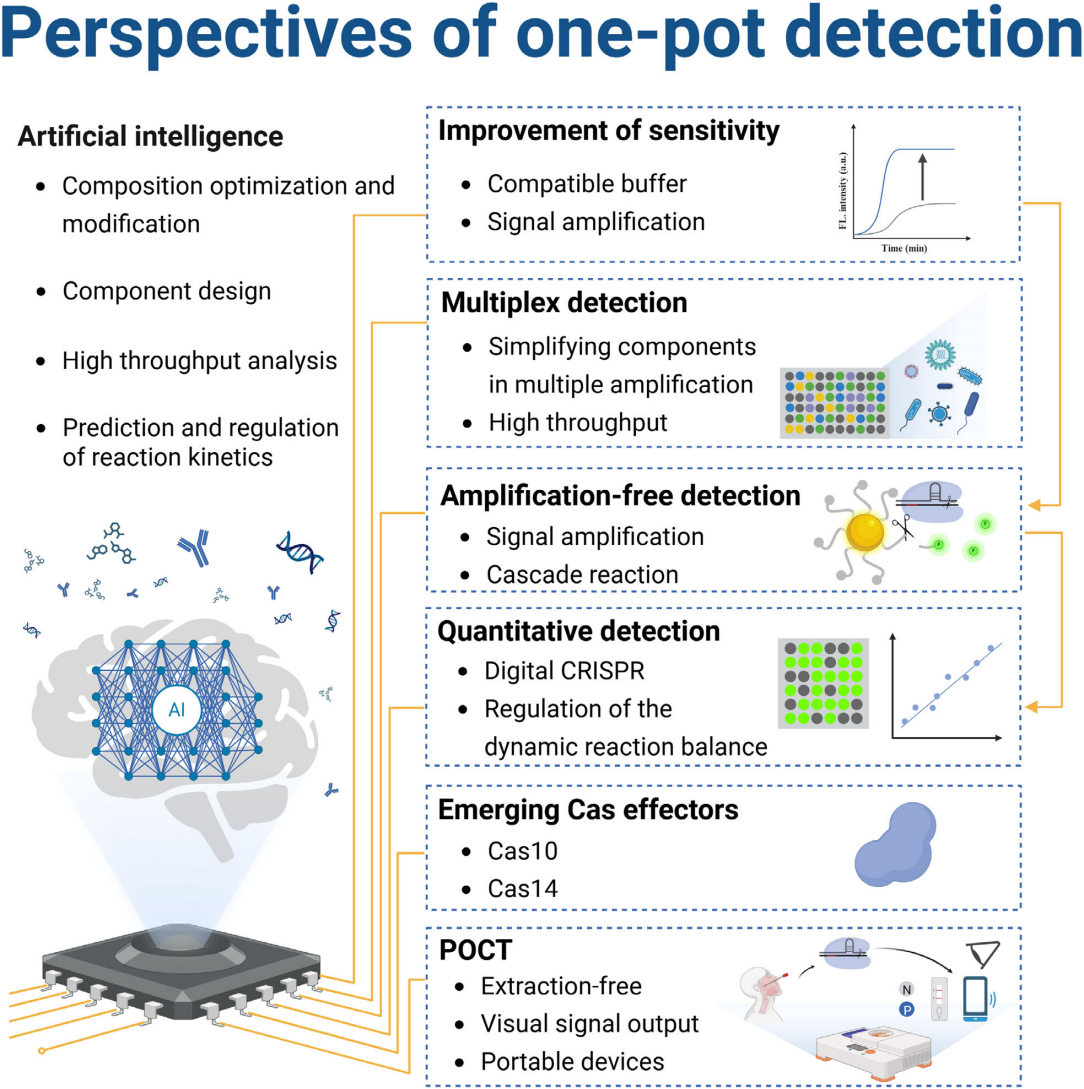
Perspectives of one‐pot CRISPR‐based detection. The key points for enhancing one‐pot assay, including improvement of sensitivity, multiplex detection, amplification‐free detection, quantitative detection, emerging Cas effectors, and POCT, which have the potential to make breakthroughs with the assistance of AI. N, negative; P, positive.

Despite significant development, the one‐pot CRISPR‐based detection strategies still have non‐negligible limitations including complex designs, high costs, and limited scalability, which requires for further innovation to break the bottleneck. The following sections outline key areas for improvement and future directions to promote one‐pot CRISPR‐based detection technologies (**Figure** **7**).

### 4.1 Improvement of Sensitivity

While existing one‐pot detection strategies have considerably addressed the incompatibility between INA and CRISPR, the sensitivity of them remains lower than that of the two‐step methods. This limits the potential of CRISPR‐based detection to be applied as a next‐generation technique for efficient diagnostics, particularly for real clinical applications, such as the diagnosis of infection with low viral load (e.g., human immunodeficiency virus) and detection of patient samples containing low‐level biomarkers (e.g., urine, cerebrospinal fluid, feces, sputum, and peritoneal fluid).^[^ ^165^ ^]^ The component of a one‐pot detection system is complex because INA and CRISPR need different buffers with different cofactors and enzymes. This incompatibility increases system complexity and has the potential for unintended biochemical inhibition, ultimately compromising detection efficiency. Developing an effective Cas protein with high cleavage activity and compatible buffers is the key point for future research to improve sensitivity. Notably, it is essential to develop kinetic models for quantitative characterization of the inhibitory effects of CRISPR cis/trans‐cleavage on INA in a one‐pot system, which is beneficial to mitigate the inhibition and enhance performance. In addition, the integration of signal amplification strategies, such as those used in amplification‐free detection including optimization of crRNAs and signal reporters, digital‐CRISPR detection platforms, signal transducer formation, and cascade signal amplification,^[^ ^29^ ^]^ can offer a promising avenue for enhancing sensitivity.

### 4.2 Multiplex Detection

Multiplex detection expands the scope of identifiable targets, which is essential for disease diagnosis, pathogen screening, and viral genotyping, and beneficial to POCT and cost‐efficiency applications. Current multiplex detection based on CRISPR utilizes various strategies, including microfluidic,^[^ ^166^ ^]^ orthogonal systems,^[^ ^167^ ^]^ lateral flow,^[^ ^168^ ^]^ and signal logic‐gate.^[^ ^169^ ^]^ These techniques typically rely on spatial isolation devices because CRISPR‐based detection systems cannot distinguish between multiple targets due to non‐specific cleavage of reporters and potential cross‐reactions. In addition, a mix of multiple primers and targets increases the complexity of the amplification system, which can lead to non‐specific amplification. These challenges make one‐pot CRISPR‐based multiplex detection particularly difficult. Some reported strategies have attempted to develop one‐pot multiplex detection by using different Cas13 effectors with orthogonal trans‐cleavage properties. For example, in one‐pot multiplexed detection for SARS‐CoV‐2 VOCs, the orthogonal trans‐cleavage activities of LwaCas13a and PsmCas13b on distinct bystander nucleic acid probes were exploited to enable simultaneous detection of multiple targets. In addition, the concentrations of reaction components were carefully optimized to ensure efficient and robust performance within a one‐pot reaction system.^[^ ^170^ ^]^ Similarly, orthogonal systems combining Cas12a/Cas13a also are employed to develop one‐pot multiplex detection due to their respective DNA and RNA substrate cleavage dependencies.^[^ ^171^, ^172^ ^]^ Despite advances in one‐pot multiplex CRISPR assays, these methods remain constrained in the number of targets they can detect simultaneously and require rigorous validation of system orthogonality across key parameters: 1) crRNA/Cas specificity, 2) target recognition, 3) fluorescence readout, and 4) reporter cleavage preferences to eliminate interference between channels.^[^ ^173^ ^]^ These limitations increase the investment of manpower and material resources, and may affect the reliability of the results because of cross cleavage. In addition, multiple enzymes and primers are collected in one tube, increasing system complexity and causing potential compromise on detection efficiency and accuracy. Currently, microfluidic chip provides an effective tool for achieving high‐throughput one‐pot multiplex detection. The POCMT system offers an instance, which can simultaneously detect 10 infectious viruses.^[^ ^153^ ^]^ However, despite the technical promise, this strategy is limited by complexity in chip design and high production costs.

The implementation of multiplex detection in CRISPR/Cas systems remains challenging due to several factors, including non‐specific collateral cleavage activity, limited signaling strategies, and the potential cross‐reactivity between targets. Multiplex CRISPR‐based one‐pot detection can be further improved by the introduction of strategies for multiplex detection, such as microfluidic spatial separation, orthogonal Cas enzymes for target‐specific recognition, bioinspired photonic crystal (PhC) barcodes,^[^ ^174^ ^]^ quantum dots,^[^ ^175^ ^]^ and novel design strategies. Further studies are essential to optimize the robustness of the system and expand the multiplexing capabilities of one‐pot CRISPR/Cas detection platforms, thereby promoting high‐throughput multiplex CRISPR‐based one‐pot detection.

### 4.3 Amplification‐Free Detection

Relatively low sensitivity of CRISPR‐based detection is often improved by coupling with INA, but this also complicates one‐pot detection. Eliminating the need for pre‐amplification of targets can simplify the development of one‐pot detection systems. Researchers are exploring amplification‐free detection methods based on CRISPR, mainly involving signal amplification and cascade reaction. For example, modified DNA reporters on lipid‐membrane‐coated microbeads could be cleaved by activated Cas12a, producing rapid signal enhancement without amplification.^[^ ^176^ ^]^ Another promising approach was the dual nucleases‐assisted cyclic amplification (DUNCAN) strategy, enabling one‐pot detection of miRNAs. This strategy combined CRISPR/Cas13a‐mediated target detection with cyclic reactions using DNA probes protected by polydopamine nanospheres for signal amplification and result readout.^[^ ^177^ ^]^ In addition, researchers have investigated the use of combined crRNAs those target different sites of the same DNA target to amplify signals.^[^ ^178^ ^]^ Recently, Lim et al. established an innovative CRISPR‐Cascade assay using an internal positive feedback loop containing two transducers, in which ribonucleoprotein (RNP) T1 was triggered by the target to trans‐cleave the blocked nucleic acids, thus generating activators that could initiate the trans‐cleavage of RNP T2 on reporters and blocked nucleic acids.^[^ ^179^ ^]^ These amplification‐free CRISPR‐Cas detection technologies have promising prospects and can pave the way for simple, rapid one‐pot detection methods that do not require pre‐amplification.

### 4.4 Quantitative Detection

Quantitative detection is critical for clinical diagnosis, especially for pathogen load quantification and the evaluation of therapeutic efficacy. However, the exponential amplification of targets in INA‐based CRISPR detection systems makes it difficult to accurately quantify the original target concentration. As a result, most one‐pot assays those have already been developed are limited to qualitative detection. Recent technological strategies have facilitated quantitative detection in one‐pot systems, such as linear fit after tuning the dynamic reaction balance in one pot,^[^ ^180^ ^]^ amplification‐free strategies, and microfluidic‐assisted digital CRISPR detection. In these approaches, only digital CRISPR allows absolute quantification of nucleic acid molecules, which is achieved by counting the number of positive units.^[^ ^154^ ^]^ More specifically, INA occurs in microreactors on microfluidic chips, where the CRISPR/Cas system cleaves reporters to generate fluorescence signals. Microfluidic digital chips, including chamber microfluidic chips and droplet microfluidic chips, have been used for this purpose. Chamber chips create thousands of picoliter‐ or nanoliter‐sized chambers, while droplet chips form closed reaction units via emulsification and dispersion.^[^ ^154^ ^]^ Recently, a droplet pairing‐merging enabled digital RPA‐CRISPR/Cas12a (DIMERIC) assay successfully achieved rapid quantification of nucleic acids within 20 min, in which a microfluidic chip with a calabash‐shaped microwell array was introduced to sequentially and size‐selectively trap large‐volume RPA droplets and small‐volume CRISPR/Cas12a droplets for generating one‐to‐one droplet pairs.^[^ ^181^ ^]^ While these technologies are promising, challenges remain, such as the immaturity of amplification‐free technology, the complexity of chamber microfluidic chip processing, and deficient droplet stability and potential background signal interference in droplet chips. Furthermore, the reliance on digital instruments makes digital CRISPR unsuitable for POCT in its current form.

### 4.5 Emerging Cas effectors

While most current CRISPR‐based diagnostics focus on Cas12 and Cas13 systems, a variety of emerging Cas enzymes are being developed in succession, such as Cas10 and Cas14, and show superior performance in stability, specificity, and anti‐interference. Similar to type VI (Cas13‐based) systems, the type III CRISPR/Cas10 system also recognizes RNA targets.^[^ ^182^, ^183^ ^]^ Significantly, unlike class 2 systems (types II, V, and VI) that rely on a single effector protein, Cas10 utilizes a multi‐subunit effector complex. This structural distinction contributes to its superior stability at room temperature, enhancing its suitability for POCT applications.^[^ ^120^ ^]^ Cas10 features two key target RNA‐activated reactions: 1) the Palm domain‐mediated synthesis of cyclic oligoadenylate (cOA), and 2) HD domain‐mediated collateral DNA cleavage.^[^ ^120^, ^182^, ^184^ ^]^ Both mechanisms have been successfully exploited for nucleic acid detection. Once activated by target RNA, Cas10 protein generates ≈1000 cOA molecules alongside protons and pyrophosphates, all of which can serve as measurable signals. Based on these outputs, a CRISPR/Cas10‐based assay has been successfully established for SARS‐CoV‐2 detection in combination with INA.^[^ ^183^ ^]^ In addition, the cOA‐dependent nonspecific RNase TTHB144 from *Thermus thermophilus* HB8 was introduced to enable cOA‐activated cleavage of reporter RNA for fluorescence signal.^[^ ^185^ ^]^ Similar to Cas13, Cas10 holds HD‐hosted collateral DNA cleavage activity triggered by identifying and binding target RNA, which has been utilized to successfully develop detection methods for medium‐length RNAs and miRNAs.^[^ ^120^, ^186^ ^]^ Compared to CRISPR/Cas13‐based detection assay, CRISPR/Cas10 offers several significant advantages: 1) the use of DNA reporters enhances signal stability; 2) the dCsm (Csm3^D34A^) mutation of the LdCsm effector complex prevents degradation of target RNA, allowing sustained cleavage of DNA reporters and reducing amplification template degradation in one‐pot detection; 3) the improved specificity due to superior tolerance to nonspecific targets enhances performance in complex clinical samples; and 4) the availability of thermophilic Cas10 variants broadens the usable temperature range, improving compatibility with INA conditions.^[^ ^120^, ^183^, ^185^, ^186^ ^]^

Similar to Cas12, Cas14 is capable of targeting and cleaving both dsDNA and ssDNA, with activation of nonspecific trans‐cleavage of ssDNA.^[^ ^187^, ^188^ ^]^ As an emerging Cas effector, Cas14 exhibits exceptionally low tolerance for mismatches, making it particularly suitable for single nucleotide polymorphism (SNP) detection.^[^ ^122^ ^]^ Therefore, Cas14 has been employed to develop a detection platform for SNP genotyping and molecular diagnostics, such as DETECTR‐Cas14 and Cas14VIDet.^[^ ^189^, ^190^ ^]^ Although Cas10 and Cas14 have not yet been introduced into one‐pot INA‐CRISPR detection systems, their distinct advantages, including template protection, improved specificity, and SNP discrimination, highlight their potential to advance one‐pot CRISPR diagnostics for POCT and novel molecular detection applications.

### 4.6 POCT Application

Currently, most one‐pot CRISPR detection strategies are still laboratory‐based and require professional instruments. In addition, nucleic acid extraction and optical instruments for result readout are often necessary because CRISPR detection typically relies on fluorescence signals. These factors complicate the use of CRISPR in POCT. To facilitate POCT, researchers have developed various simple and rapid pretreatment methods of samples to eliminate the need for extraction. For example, Qiu et al. developed an extraction‐free, one‐pot CRISPR‐assisted detection (ExCad) method that included a rapid pretreatment process for sputum samples.^[^ ^191^ ^]^ However, the universality of such methods still needs further validation. Microfluidics has also been employed to integrate the entire detection process, from sample processing to signal reading. Visual detection methods, such as colorimetry and lateral flow assays, offer intuitive results, but lateral flow assays may suffer from cross‐contamination due to the open operation condition.

Portable devices that enable process automation and sample‐in‐answer‐out testing have been developed. For instance, Chen et al. proposed a one‐pot CRISPR‐based “Green‐Yellow‐Red” multiplex detection strategy integrated into a portable cartridge, where results were automatically analyzed by custom software.^[^ ^69^ ^]^ Similarly, Lee et al. designed an automated assay system that integrated a rotating sample holder, heating components, an optical detection module, and microcontroller units for on‐site HPV testing.^[^ ^192^ ^]^ The development of portable integrated instruments, such as SIMPLEone, has also enabled one‐pot detection of SARS‐CoV‐2 and other respiratory viruses using a custom mobile phone application.^[^ ^193^ ^]^ These portable devices, which integrate the entire process from sample processing to result output without the need for professional instruments, hold great promise for enabling multiplex detection. The advanced automation significantly minimizes the requirements for professional operation, thus optimizing the accessibility of the operation for non‐trained operators. However, the high production costs associated with modern diagnostic platforms, combined with their complicated manufacturing protocols, continue to pose significant techno‐economic constraints that prevent widespread clinical use.

### 4.7 Artificial Intelligence Assistance

AI, which encompasses machine learning and deep learning paradigms, has become a groundbreaking technological innovation with wide‐ranging applications in numerous fields, particularly in biomolecular sciences and healthcare.^[^ ^194^, ^195^, ^196^, ^197^, ^198^ ^]^ The complexity of component optimization and modification, device design, prediction and regulation of reaction kinetics, and massive data analysis associated with the one‐pot CRISPR‐based detection strategy pose barriers to its application, which are expected to be addressed by AI. So far, AI has successfully assisted CRISPR technology in areas such as thermostability prediction of Cas,^[^ ^199^ ^]^ superior crRNA design,^[^ ^200^ ^]^ activity prediction,^[^ ^201^ ^]^ excavation of Cas,^[^ ^202^ ^]^ high‐throughput detection results processing,^[^ ^203^ ^]^ and microfluidic design.^[^ ^204^ ^]^ Although AI‐assisted CRISPR is mainly focused on gene editing applications, it is now spreading to the field of molecular diagnostics with considerable momentum. In addition, researchers realized mutation design to improve enzyme thermostability with AI,^[^ ^205^ ^]^ further confirming the feasibility of AI‐assisted one‐pot CRISPR assay. Accordingly, AI is potential to support various scenarios in development of the one‐pot CRISPR‐based detection platform, including: 1) the efficient design of detection components and integrated microfluidic devices, 2) the optimization of reaction conditions through predictive activity modeling, 3) the development of thermostable Cas variants through computational protein engineering and stability prediction algorithms, 4) standardized and automated results analysis, and 5) the development of Cas activity regulator by predicting reaction kinetics.

As an emerging technology, CRISPR offers significant potential for nucleic acid detection, with applications in diagnostics, pathogen monitoring, and genetic analysis. However, there are still several challenges in practical applications, particularly when coupled with INA to fulfill sensitivity requirements for real‐world diagnostic scenarios. One‐pot detection systems, which address issues introduced by the two‐step INA‐CRISPR assay such as aerosol contamination, multi‐step workflows, and system incompatibilities, are crucial to the widespread adoption of CRISPR‐based technologies. Despite the considerable progress, further innovations are required to overcome existing barriers and unlock the full potential of CRISPR for both multiplex and quantitative detection. The ongoing development of robust, scalable, and cost‐effective CRISPR‐based detection systems holds promise for revolutionizing the future of nucleic acid diagnostics.

## Conflict of Interest

The authors declare no conflict of interest.
